# Biochemical properties and subcellular localization of six members of the HXK family in maize and its metabolic contribution to embryo germination

**DOI:** 10.1186/s12870-018-1605-x

**Published:** 2019-01-15

**Authors:** Giovanna Paulina Aguilera-Alvarado, Ángel Arturo Guevara-García, Samuel Abraham Estrada-Antolín, Sobeida Sánchez-Nieto

**Affiliations:** 1 Departamento de Bioquímica, Facultad de Química, Conjunto E., Universidad Nacional Autónoma de México, CDMX, Mexico; 2 Departamento de Biología Molecular de Plantas, Instituto de Biotecnología, Universidad Nacional Autónoma de México, Cuernavaca, Mexico

**Keywords:** Hexokinase, Maize, Germination, Biochemical characterization, Cytosolic HXK, Mitochondrial hexokinase

## Abstract

**Background:**

Seed germination is a crucial process in the plant life cycle when a dramatic variation of type and sugar content occurs just as the seed is hydrated. The production of hexose 6 phosphate is a key node in different pathways that are required for a successful germination. Hexokinase (HXK) is the only plant enzyme that phosphorylates glucose (Glc), so it is key to fueling several metabolic pathways depending on their substrate specificity, metabolite regulatory responses and subcellular localization. In maize, the HXK family is composed of nine genes, but only six of them (ZmHXK4–9) putatively encode catalytically active enzymes. Here, we cloned and functionally characterized putative catalytic enzymes to analyze their metabolic contribution during germination process.

**Results:**

From the six HXKs analyzed here, only ZmHXK9 has minimal hexose phosphorylating activity even though enzymatic function of all isoforms (ZmHXK4–9) was confirmed using a yeast complementation approach. The kinetic parameters of recombinant proteins showed that ZmHXK4–7 have high catalytic efficiency for Glc, fructose (Fru) and mannose (Man), ZmHXK7 has a lower Km for ATP, and together with ZmHXK8 they have lower sensitivity to inhibition by ADP, G6P and N-acetylglucosamine than ZmHXK4–6 and ZmHXK9. Additionally, we demonstrated that ZmHXK4–6 and ZmHXK9 are located in the mitochondria and their location relies on the first 30 amino acids of the N-terminal domain. Otherwise, ZmHXK7–8 are constitutively located in the cytosol. HXK activity was detected in cytosolic and mitochondrial fractions and high Glc and Fru phosphorylating activities were found in imbibed embryos.

**Conclusions:**

Considering the biochemical characteristics, location and the expression of ZmHXK4 at onset of germination, we suggest that it is the main contributor to mitochondrial activity at early germination times, at 24 h other ZmHXKs also contribute to the total activity. While in the cytosol, ZmHXK7 could be responsible for the activity at the onset of germination, although later, ZmHXK8 also contributes to the total HXK activity. Our observations suggest that the HXKs may be redundant proteins with specific roles depending on carbon and ATP availability, metabolic needs, or sensor requirements. Further investigation is necessary to understand their specific or redundant physiological roles.

**Electronic supplementary material:**

The online version of this article (10.1186/s12870-018-1605-x) contains supplementary material, which is available to authorized users.

## Background

Seed germination, which starts with the imbibition, is a crucial event in the plant life cycle when metabolic activity resumes, and reserves are mobilized to support initial plant development. These are key steps to sustain the seedling growth before photosynthetic machinery lights up [1]. After germination, high content of hexoses 6 phosphate was detected in wild oat, *Arabidopsis* and rice seeds [2, 3], which indicates the activation of metabolism. Soluble sugars support the metabolic activity at the onset of germination, followed by the massive degradation of starch reserves and even the components of the cell wall at later times [1, 2, 4, 5]. Sugars are the main source of carbon and energy, but they also have signaling functions [6]. Therefore, the dramatic changes of sugar type and concentration during the germination process undoubtedly regulates the enzyme activity and the pattern of gene expression of several enzymes [5, 6].

Hexokinase (HXK) is a glycolytic regulatory enzyme that catalyzes the irreversible phosphorylation reaction of D-hexoses at the sixth carbon using ATP-Mg^2+^ as a phosphate donor. The HXK family members, that can phosphorylate several hexoses, including glucose (Glc), mannose (Man) and fructose (Fru) [7], can be localized at cytosol, mitochondria, nucleus or chloroplast depending of the presence of an anchor or signal peptide at their N-terminal sequence [8, 9]. The metabolic flux of substrates through different pathways, like the tricarboxylic acid cycle (TCA), oxidative pentose phosphate pathway (OPPP), the fatty acid synthesis and nucleoside diphosphate sugar biosynthesis, depends on the localization of the HXK’s metabolic products and other downstream metabolic intermediates [7, 9, 10]. Furthermore, inhibition of germination in fenugreek, mung beans, white mustard and wheat seeds, was detected with HXK substrates like 2-deoxyglucose, D-mannose (Man), and D-glucosamine [11]. This, along with the low rate of germination of *oshxk7* null mutants under O_2_-deficient conditions [12], reveals the key role that HXK has in providing energy and carbon skeletons to different pathways during this demanding process. Therefore, the expression of different HXKs at specific times and subcellular locations may contribute to control the metabolic flux during the germination.

Regardless of its catalytic function, a substantial amount of research demonstrated that HXKs function as Glc sensors in plants. For example, in response to Glc abundance, HXK promotes the suppression of some photosynthetic genes, including the rubisco small subunit, carbonic anhydrase, sedoheptulose bisphosphatase, and the chlorophyll a/b-binding protein [13–16]. Although HXKs are the only enzymes that phosphorylate Glc in plants [7], their catalytic activity has received less attention than their Glc sensor function [8]. Only a few HXKs has been characterized so far, some of them in subcellular fractions that could very well contain several HXKs [17–24]. We believe that due to its potential to eventually manipulate plant productivity, the study of hexokinase deserves much more attention. Unfortunately, the study of HXK families particularly in important crops is incipient in many ways.

In maize the HXK family is composed of nine genes. Six of them (ZmHXK4–9) putatively encode catalytically active HXKs. The other three (*ZmHXK3a*, *ZmHXK3b* and *ZmHXK10*), according to their sequence homology, encode proteins structurally similar to HXKs, but lacking catalytic activity, they are called HXK-like (HXL) [25, 26]. HXK activity has been detected in cytosolic, mitochondrial and Golgi membrane fractions of maize roots [21, 27, 28]. However, based in gene sequence analysis has been suggested that maize HXKs might be located in the cytosol, mitochondria and chloroplast membranes [25, 26]. Thus, we understand that the subcellular location and the number of enzymes that contribute to the total hexokinase activity in each cell compartment require clarification. Then the contribution each maize HXKs during a particular physiological process could be addressed. Elucidate the participation of a protein in any metabolic or developmental processes is difficult when mutants are not available. However, we hypothesize that the possible contribution of the six putative catalytic maize HXKs during the germination process, could be studied comparing their expression patterns, their catalytic parameters and they subcellular location. Besides, our study provides detailed molecular information on six HXKs that differentiate themselves by substrate specificity, metabolite regulatory responses and subcellular localization. This information helps us to understand how different HXKs could be involved in a physiological process like maize seed germination.

## Results

### Identifying the maize HXK family members that have catalytic activity by in silico analysis

To study the HXK maize isoenzymes that contribute to the metabolism on the germination process, and their evolutionary relationships between HXK protein families, 112 HXKs plant protein sequences were aligned using MEGA X [29]. The evolutionary history was inferred by using the Maximum Likelihood method based on the JTT matrix-based model [30, 31] using 2000 bootstrap. The phylogenetic analysis included two yeast HXKs sequences as an out-group (see Additional file 1: Figure S1 and Table S1). Since some HXKs are already characterized, we found that the HXKs were clustered according to some specific characteristics such as subcellular localization or lack of HXK activity. The maize HXKs were found in three clusters in the cladogram tree, ZmHXK7 and ZmHXK8 are related to the monocot HXKs, such as OsHXK7 and OsHXK8 [12, 25, 32, 35]. Apparently, the mitochondrial HXKs are grouped in three subgroups: one mitochondrial HXKs in dicot plants, and two subgroups of HXKs in monocots, in the latter we found four ZmHXKs; ZmHXK4, 5 and 6 are in a subgroup different to the subgroup in which ZmHXK9 is. ZmHXK3a, ZmHXK3b, and ZmHXK10 are in the Hexokinase-like (HKL) group.

**Fig. 1:**
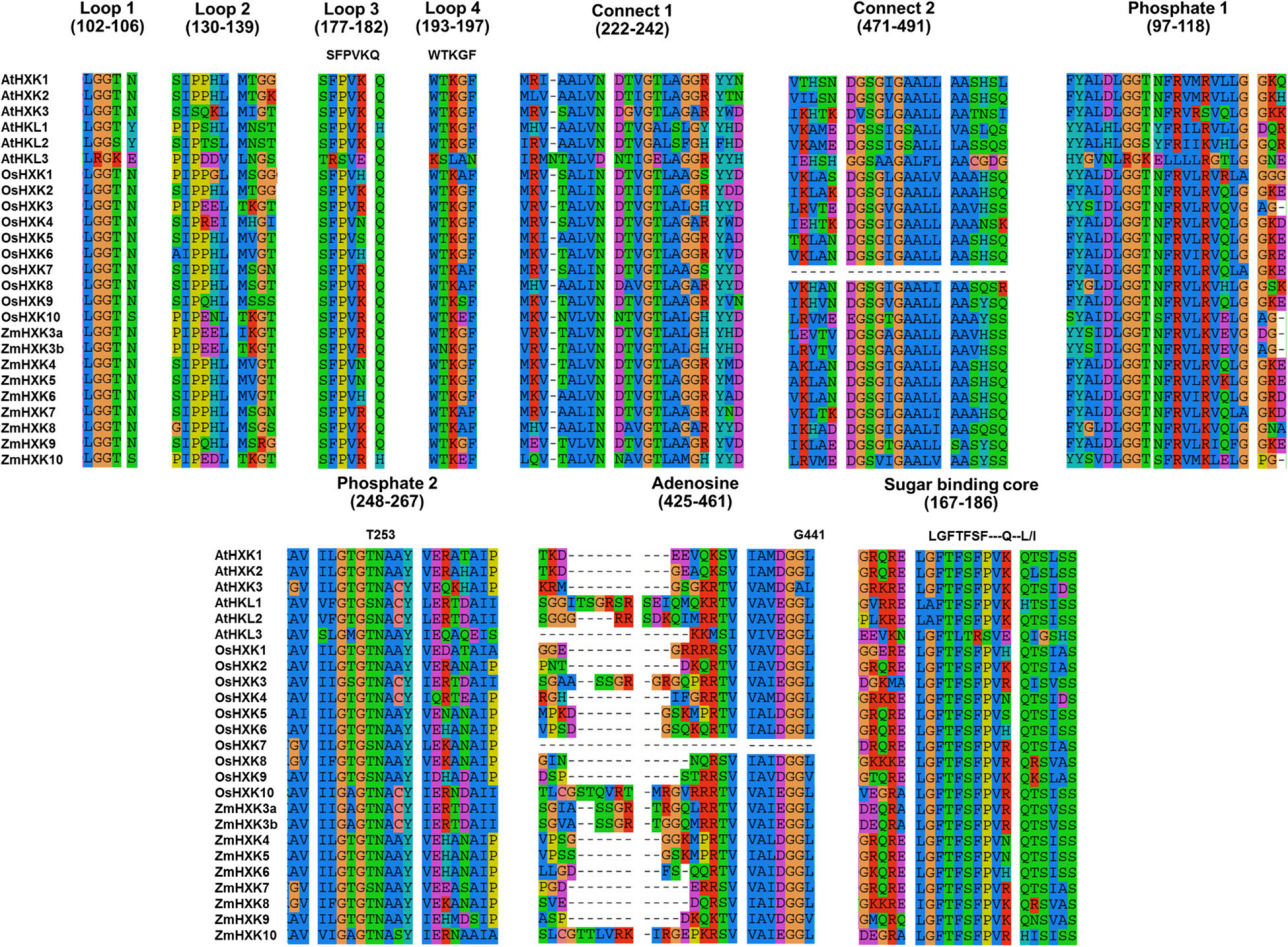
Multiple amino acid sequence alignment of several conserved binding core domains, important to the HXK catalysis in *Arabidopsis*, rice and maize HXKs. The sequences were aligned using SeaView 4 [33]. The sites involved in the adenosine and sugar binding were identified by inspection

The protein sequences of *Zea mays*, *A. thaliana* and *Oryza sativa* were further analyzed trying to visualize the amino acid differences between the putative HXK and HKL. The sequences were aligned using SeaView 4 [33] and information related to the tridimensional structure of AtHXK1 was used in the sequence analysis [20, 32]. A high degree of amino acid conservation in the putative domains, regions and conserved amino acids involved in binding substrates was identified in ZmHXK4, ZmHXK5, ZmHXK6, ZmHXK7, ZmHXK8 and ZmHXK9 (Fig. 1, see Additional file 1: Table S2). In contrast, ZmHXK3a, ZmHXK3b and ZmHXK10, have several differences in the domains and residues that are involved in substrate binding in loop 2 and adenosine binding domain (Fig. 1, see Additional file 1: Table S2). All these modifications are also found in *A. thaliana* and rice HKL proteins, which lack catalytic activity [25, 32], suggesting that ZmHXK3a, ZmHXK3b and ZmHXK10 are mostly likely HKL. Since the main goal of this work was to characterize the members of the HXK family in maize contributing to the seed germination metabolism, we focus on to clone the open reading frames (ORFs) of ZmHXK4, ZmHXK5, ZmHXK6, ZmHXK7, ZmHXK8 and ZmHXK9, and they were analyzed further.

### In vitro kinetic characterization of maize HXKs

**Fig. 2:**
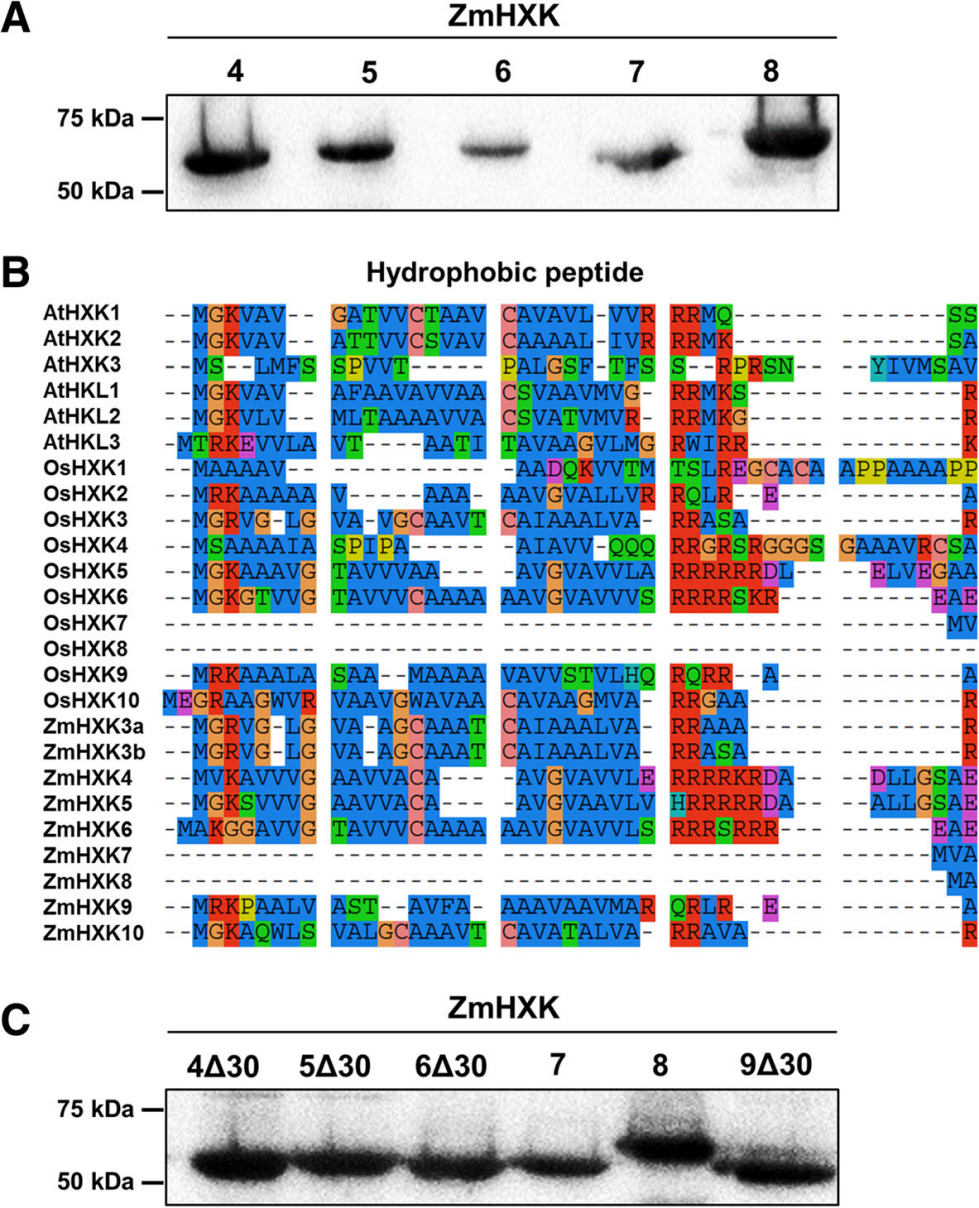
Purified recombinant full and truncated protein versions of maize HXKs, ZmHXK4–9, and the comparison of the N-terminal sequences of the HXKs from *A. thaliana*, rice and maize. **a** Western blot of purified recombinant full HXKs (**b**) N-terminal sequences from HXKs from *A. thaliana*, rice and maize. The sequence analysis was made by inspection with the aid of the software SeaView [33]. The hydrophobic sequence is approximately 20 amino acids long in several ZmHXKs and it is highly conserved among other HXKs. **c** Western blots of full and truncated HXK versions that were used to determine the kinetic parameters. The recombinant proteins have a V5 epitope which was detected using the anti-V5-HRP (R961–25, Invitrogen)

To evaluate and characterize the activity of maize HXK isoenzymes the cognate ORFs were cloned and, recombinant proteins were produced in bacteria (Fig. 2a, see Additional file 1: Figure S2). ZmHXK7 and ZmHXK8 are soluble proteins, and their kinetic parameters were directly determined. ZmHXK4–6 proteins were found in the pellet and ZmHXK9 was not produced in *E. coli*. Insoluble ZmHXK4–6 proteins were detergent solubilized and renatured, but the enzymes recovered from this process did not show HXK activity. Since some HXKs have a hydrophobic peptide that could make the enzymes insoluble, a new version of each protein was made deleting the first 30 amino acids to exclude the highly hydrophobic sequence (Fig. 2b-c). When expressed in *E. coli*, these truncated variants of ZmHXK4Δ30, ZmHXK5Δ30, ZmHXK6Δ30, and ZmHXK9Δ30, were recovered and purified from the supernatant (Fig. 2c, see Additional file 1: Figure S3), then used to determine their kinetic parameters.

**Fig. 3:**
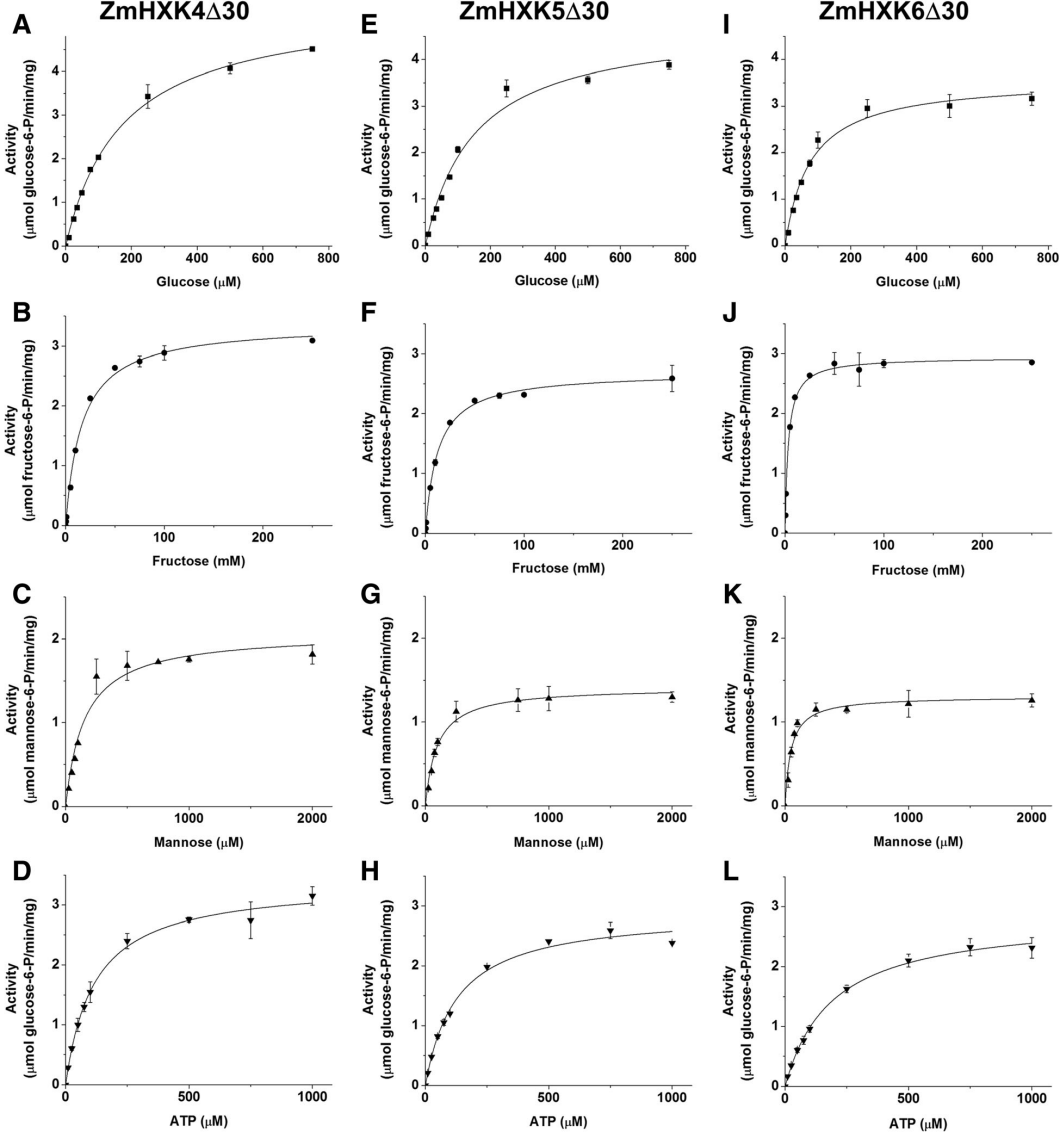
Saturation curves of HXK activity of ZmHXK4Δ30, ZmHXK5Δ30 and ZmHXK6Δ30 for glucose, fructose, mannose and ATP. HXK activity of maize isoenzymes was determined for the recombinant truncated versions: **a**, **b**, **c**, **d** ZmHXK4Δ30, (**e**, **f**, **g**, **h**) ZmHXK5Δ30, (**i**, **j**, **k**, **l**) ZmHXK6Δ30. The reaction medium contained 2.5 μg of purified recombinant protein. The effect of increasing concentrations of Glc (**a**, **e**, **i**), Fru (**b**, **f**, **j**) and Man (**c**, **g**, **k**) was determined with a fixed concentration of 2 mM ATP. To determine the Km for ATP (**d**, **h**, **l**) 5 mM Glc was included in the reaction medium. The data was adjusted to Michaelis-Menten equation. The graphics were obtained with the Origin 8.0 software. Values represent the average ± standard deviation of two technical repeats from three independent replicates

**Fig. 4:**
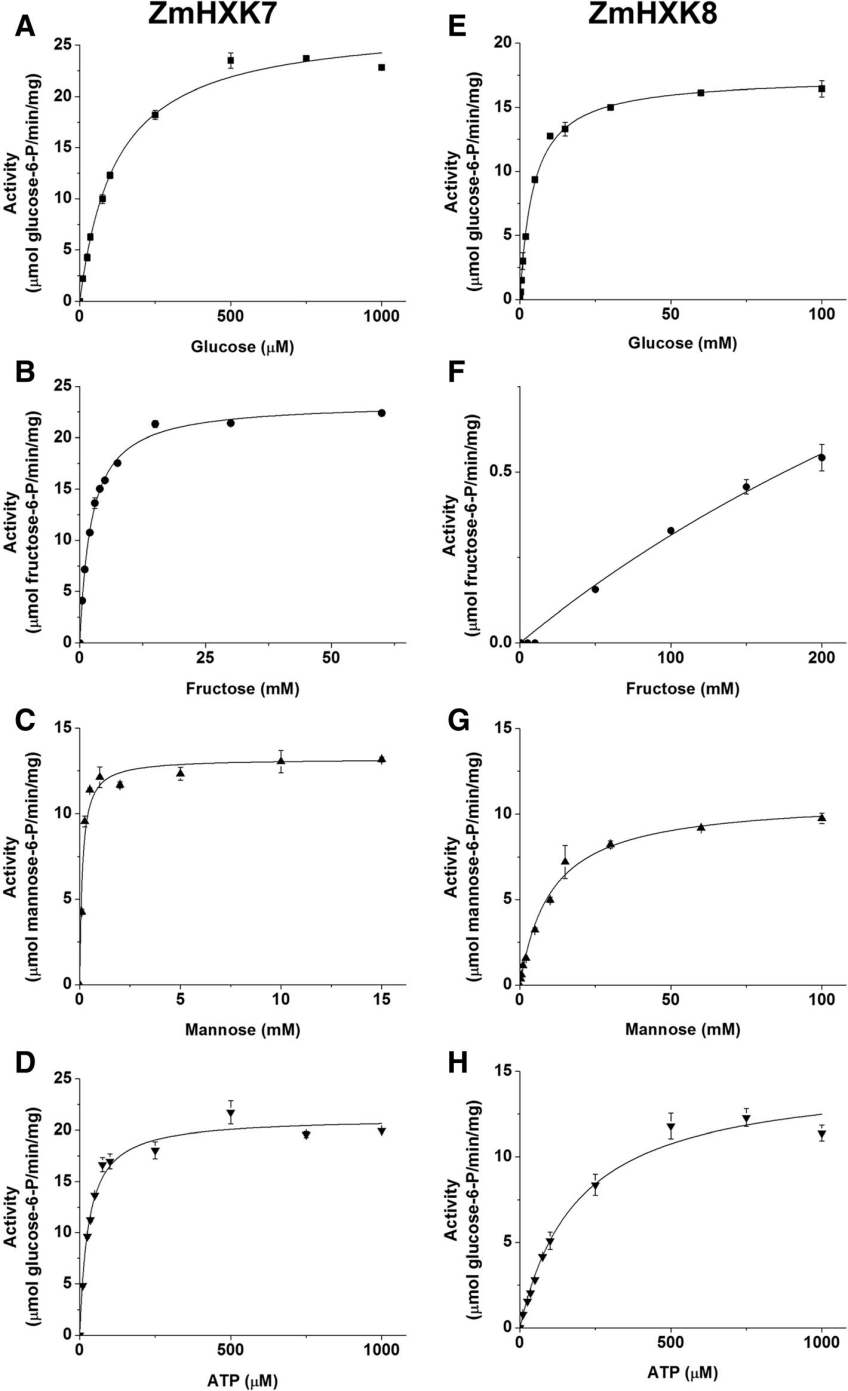
Saturation curves of HXK activity of ZmHXK7 and ZmHXK8 for glucose, fructose, mannose and ATP. The HXK activity of recombinant proteins (**a**, **b**, **c**, **d**) ZmHXK7 and (**e**, **f**, **g**, **h**) ZmHXK8 were assayed in a reaction medium with a fixed concentration of ATP (2 mM) and increasing concentration of Glc (A, E), Fru (B, F) and Man (C y G). To determine the Km for ATP (D, H) 5 mM Glc for ZmHXK7 and 50 mM Glc for ZmHXK8 were included in the reaction medium. The data was adjusted to Michaelis-Menten equation, except for ZmHXK8 which did not reach the saturation with fructose as substrate even in high concentrations. The graphics were obtained with the Origin 8.0 software. Values represent the average ± standard deviation of two technical repeats from three independent replicates

ZmHXK9Δ30 was the only protein that had minimal hexose phosphorylating activity, close to the blank, even in presence of high concentrations of each substrate, or 4 times more protein than the used to determine the activity of other HXKs, therefore the kinetic parameters were not obtained for this protein. The activity data for ZmHXK4–8 were adjusted to Michaelis–Menten kinetics for the three assayed hexoses and ATP (Figs. 3 and 4). The kinetic parameters were calculated by a non-linear regression fit (Table 1). The substrate preferences for all the maize HXKs were similar, especially for ZmHXK4Δ30-ZmHXK6Δ30. They had a smaller *K*_m_ value for Man followed by the Glc *K*_m_ value, but the lower *V*_max_ found was displayed for Man.

**Table 1:** Kinetic parameters of maize HXKs

Protein	Parameter^a^	Substrate			
		Glucose	Fructose	Mannose	ATP
ZmHXK4Δ30	*V*_max_	5.65 ± 0.12	3.05 ± 0.46	3.23 ± 0.22	1.83 ± 0.35
	*K*_m_	195.70 ± 31.96	19,324.15 ± 3914.89	138.85 ± 22.42	134.90 ± 25.74
	*k*_cat_ */K*_m_	2.85 × 10^4^	1.55 × 10^2^	2.29 × 10^4^	1.34 × 10^4^
ZmHXK5Δ30	*V*_max_	4.55 ± 0.34	2.49 ± 0.29	2.77 ± 0.19	1.54 ± 0.56
	*K*_m_	136.65 ± 20.01	30,216.8. ± 5708.76	119.55 ± 33.30	135.60 ± 11.46
	*k*_cat_/K_m_	3.27 × 10^4^	81.00	2.28 × 10^4^	1.12 × 10^4^
ZmHXK6Δ30	*V*_max_	3.60 ± 0.14	2.61 ± 0.47	3.08 ± 0.33	1.08 ± 0.32
	*K*_m_	65.86 ± 15.19	4291.84 ± 1410.04	48.85 ± 1.06	198.85 ± 12.80
	*k*_cat_ */K*_m_	5.40 × 10^4^	6.01 × 10^2^	6.23 × 10^4^	5.36 × 10^3^
ZmHXK7	*V*_max_	27.19 ± 0.77	23.44 ± 0.30	21.33 ± 0.54	13.21 ± 0.48
	*K*_m_	108.10 ± 17.96	2701.85 ± 590.93	123.79 ± 11.61	28.74 ± 0.30
	*k*_cat_ */K*_m_	2.46 × 10^5^	8.48 × 10^3^	1.68 × 10^5^	4.49 × 10^5^
ZmHXK8	*V*_max_	17.38 ± 0.24	NC	14.79 ± 0.99	10.91 ± 0.34
	*K*_m_	4687 ± 299.53	NC	10,396.50 ± 54.31	147.20 ± 62.08
	*k*_cat_ */K*_m_	3.66 × 10^3^	NC	1.40 × 10^3^	7.32 × 10^4^

^a^*V*_max_ is expressed in μmol hexose-6-P min^− 1^ mg^− 1^; *K*m is expressed in μM and *k*_cat_*/K*_m_ in M^− 1^ s^− 1^; NC: Non-calculated since the enzyme does not reach the saturation

To obtain the *K*_m_ for hexoses the ATP concentration was fixed at 2 mM. To obtain the *K*_m_ for ATP, Glc was used at a fixed concentration of 5 mM except for ZmHXK8 that was fixed at 50 mM Glc. The kinetic parameters were obtained with Origin 8.0 software and the values represent the average ± standard deviation of two technical repeats from three independent replicates

The enzyme with the smallest *K*_m_ for Glc was ZmHXK6Δ30. Moreover, ZmHXK7 had the smallest *K*_m_ for ATP and Fru, as opposed to ZmHXK8, for which it was not possible to estimate the *K*_m_ for Fru, because the enzyme did not reach saturation for this substrate (Fig. 4). ZmHXK8 had the largest *K*_m_ for Glc and Man compared to the other HXKs (Table 1).

Both products of the HXK reaction, ADP and G6P, modulate the HXK activity. It was found that ZmHXK7 and ZmHXK8 were less sensitive to ADP and G6P inhibition than ZmHXK4Δ30-6Δ30 (Table 2, Additional file 1: Fig. S4). ADP and N-acetyl-glucosamine (NAG), produced strong inhibition of the mitochondrial HXK activity in maize roots but did not alter the cytosolic activity [21, 27, 34], suggesting that ZmHXK4Δ30-6Δ30 are mitochondrial enzymes.

**Table 2:** IC_50_ for ADP, NAG and G6P for maize HXK activity

	IC_50_ (mM)		
	ADP	NAG	G6P
ZmHXK4Δ30	0.71 ± 0.15	52.14 ± 16.42	47.49 ± 2.89
ZmHXK5Δ30	0.69 ± 0.15	46.80 ± 1.82	47.46 ± 1.44
ZmHXK6Δ30	0.72 ± 0.14	25.15 ± 2.25	53.54 ± 2.48
ZmHXK7	6.47 ± 1.14	15.01 ± 3.24	IS
ZmHXK8	3.12 ± 1.14	37.54 ± 1.63	IS

*IS* Insensitive, since the HXK activity was similar to the control

HXK activity was determined using 5 mM Glc and 2 mM ATP, except for ZmHXK8 in which 50 mM Glc was used. The half maximal inhibitory values (IC_50_) were obtained with Origin 8.0 software and the values represent the average ± standard deviation of two technical repeats from three independent replicates

### The putative maize HXKs are functional enzymes

**Fig. 5:**
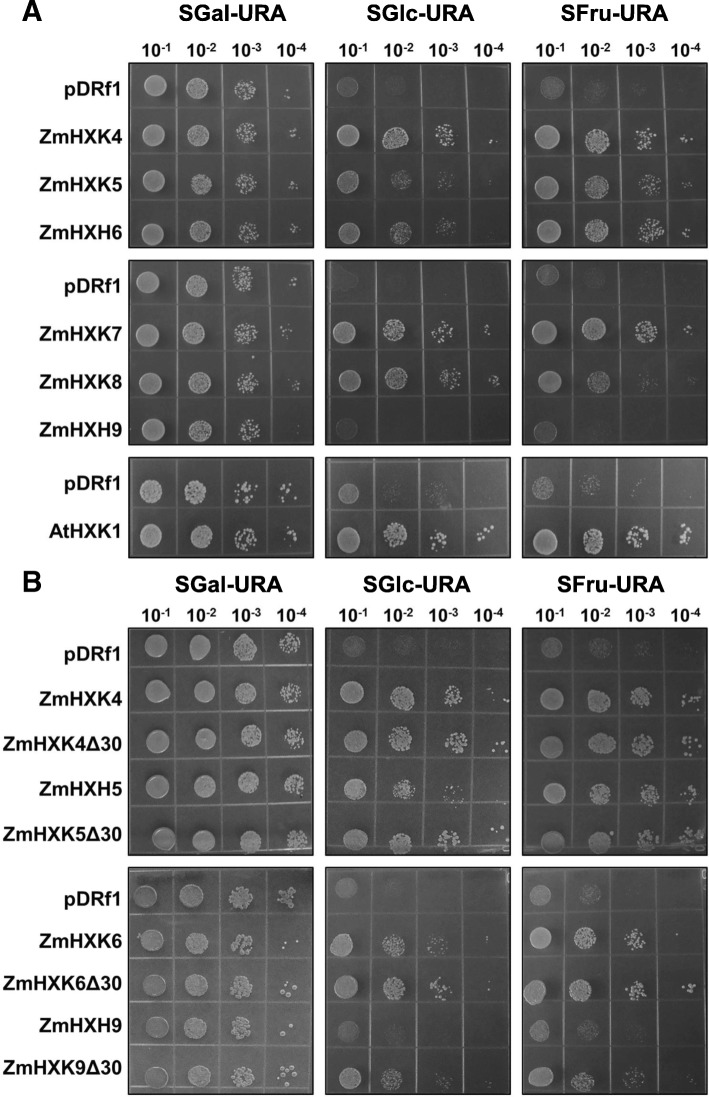
HXK activity complementation assays on yeast with native and truncated versions of maize HXKs. **a** Yeast complementation with full HXK versions. **b** Yeast complementation with truncated HXK versions. Four different serial dilutions of yeast cultures were plated in a selective medium with three carbon sources: Gal (SGal-URA), Glc (SD-URA) and Fru (SFru-URA). Negative control was made with yeast strain transformed with the empty vector (pDRf1), and the positive control consisted of the yeast strain transformed with *AtHXK1*. Representative images of three independent replicates

A yeast complementation assay was used to verify the enzymatic activity of ZmHXK4–9 (without any tag) in vivo. For this assay, we used the JT 20088 mutant of *S. cerevisiae* that has disrupted *hxk1, hxk2 and glk1* genes and it cannot use Glc and Fru as carbon sources (galactose was used as the carbon source). Only ZmHXK9 could not complement the yeast mutant, suggesting its lack of HXK activity or poor recombinant protein expression, however a PCR reaction to determine the expression profile of each HXK in the yeast mutant showed a low expression of ZmHXK9 (Additional file 1: Figure S5). Conversely, yeast growth was possible in Glc and Fru supplemented media for cells transformed with the ZmHXK4–8 genes, although some differences were found (Fig. 5a). The yeast carrying full versions of the ZmHXK5 and ZmHXK6 genes grew slower in Glc medium than carrying the ZmHXK4 gene. In *E. coli* ZmHXK4Δ30-6Δ30 have almost similar *K*_m_ among them (Table 1), we speculate that in the full versions the N-terminal domain could have a negative effect on the enzyme activity when expressed in yeast, as full version proteins are insoluble in *E. coli* and in the case of ZmHXK9 the sequence of the N-terminal could affect the transcript level. To explore these possibilities, a yeast complementation assay was performed with ZmHXK4Δ30-6Δ30 and ZmHXK9Δ30. All yeast transformed with the truncated maize HXK versions have a high level of *ZmHXK* expression even in ZmHXK9Δ30 (see Additional file 1: Figure S5). Yeast mutant expressing ZmHXK4Δ30 and ZmHXK6Δ30 grew better than those that expressed the full versions (Fig. 5b), even ZmHXK9Δ30 complemented the yeast mutant. All together, these results show that the six maize HXKs analyzed here are functional enzymes and support the idea that the N-terminal domain affects the enzymatic activity in yeast at least for ZmHXK5 and ZmHXK6 (Fig. 2c). In the case of ZmHXK9 the N-terminal affects its transcription in the mutant yeast.

### Prediction of the subcellular localization

A different subcellular localization was proposed for the maize HXKs. According to the cladogram tree, the maize HXKs are predicted to be at the mitochondria and cytosol (see Additional file 1: Figure S1). Since the subcellular localization of an enzyme is determinant for its physiological function, we identified the amino acid sequence using the TargetP 1.1 server [32] that predicts the localization of active HXK members in maize. ZmHXK4, ZmHXK5, ZmHXK6, and ZmHXK9 have a large hydrophobic domain (Fig. 2c) and is predicted to be a transmembrane domain which is could be used to secrete the protein (Addtional file 1: Table S3). Proteins with similar helical domains that attach proteins to the outer mitochondrial membrane were found in AtHXK1, AtHXK2, OsHXK5, and OsHXK6 (Fig. 2c) [33, 36–40], suggesting that ZmHXK4, ZmHXK5, ZmHXK6, and ZmHXK9 are outer mitochondrial membrane proteins. By contrast, ZmHXK7 and ZmHXK8 do not have sequences that could act as transit peptides or transmembrane domains (Fig. 2c and Additional file 1: Table S3). Similar results were obtained for the cytosolic HXKs of rice, OsHXK7 and OsHXK8 [12, 37], suggesting that ZmHXK7 and ZmHXK8 could be in the cytosol. This is possible even if the TargetP 1.1 software [35] predicted that ZmHXK8 was located at the chloroplast.

### Expression of maize active HXKs fused to green fluorescent protein (GFP)

**Fig. 6:**
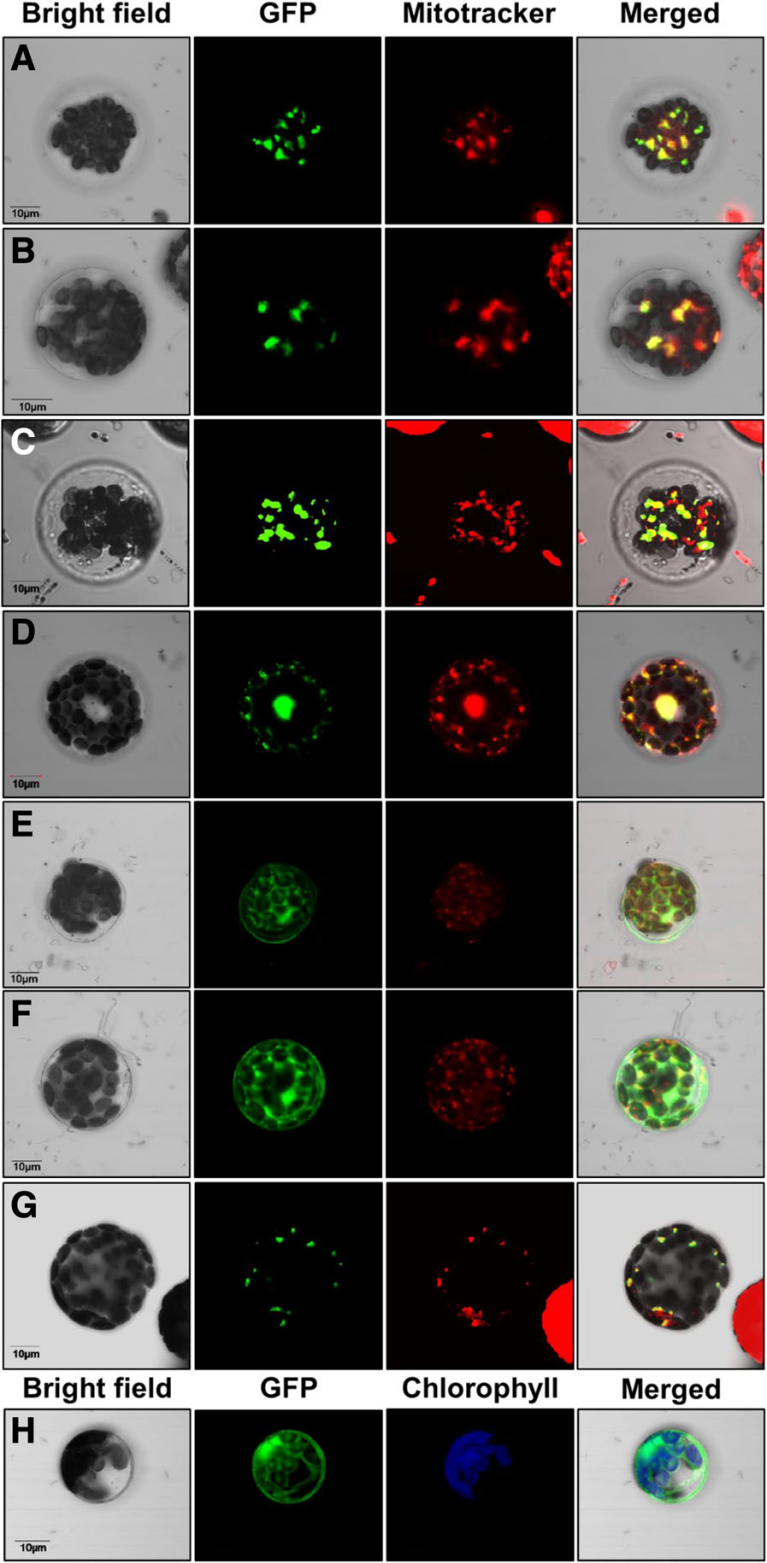
Maize HXKs are localized in the mitochondria and the cytosol. Zeta-projection images of protoplasts from *A. thaliana* transformed with the full version of (**a**) ZmHXK4, (**b**) ZmHXK5, (**c**) ZmHXK6, (**d**) ZmHXK9, (**e**) ZmHXK7, (**f**) ZmHXK8 translationally fused at the GFP carboxy-terminus are shown. As a reference, the fluorescence of GFP alone (**h**) and AtHXK1 fused at GFP (**g**) are shown. Mitotracker was used as mitochondrial marker, and it is shown in red; chlorophyll autofluorescence was used as marker of plastid localization and it is shown in blue. The merge corresponds to signals of bright field, GFP and Mitotracker channel. Bar = 10 μm. Representative images of four independent replicates

To establish the intracellular localization of the maize HXKs, a transient expression system using *A. thaliana* protoplasts transformed with ZmHXK4–9:GFP fusion proteins was analyzed. As reference of mitochondrial localization, Mitotracker dye staining intact mitochondria was monitored. GFP fluorescence from ZmHXK4:GFP, ZmHXK5:GFP, ZmHXK6:GFP, and ZmHXK9:GFP co-localized with the orange Mitotracker signal (Fig. 6a-d), similar to AtHXK1-GFP (Fig. 6g) indicating that these proteins are target to mitochondria. ZmHXK7:GFP and ZmHXK8:GFP apparently accumulate in the cytoplasm (Fig. 6e-f), similar to the fluorescence observed with the GFP alone (Fig. 6h).

### The N-terminus directs the protein to the mitochondria

**Fig. 7:**
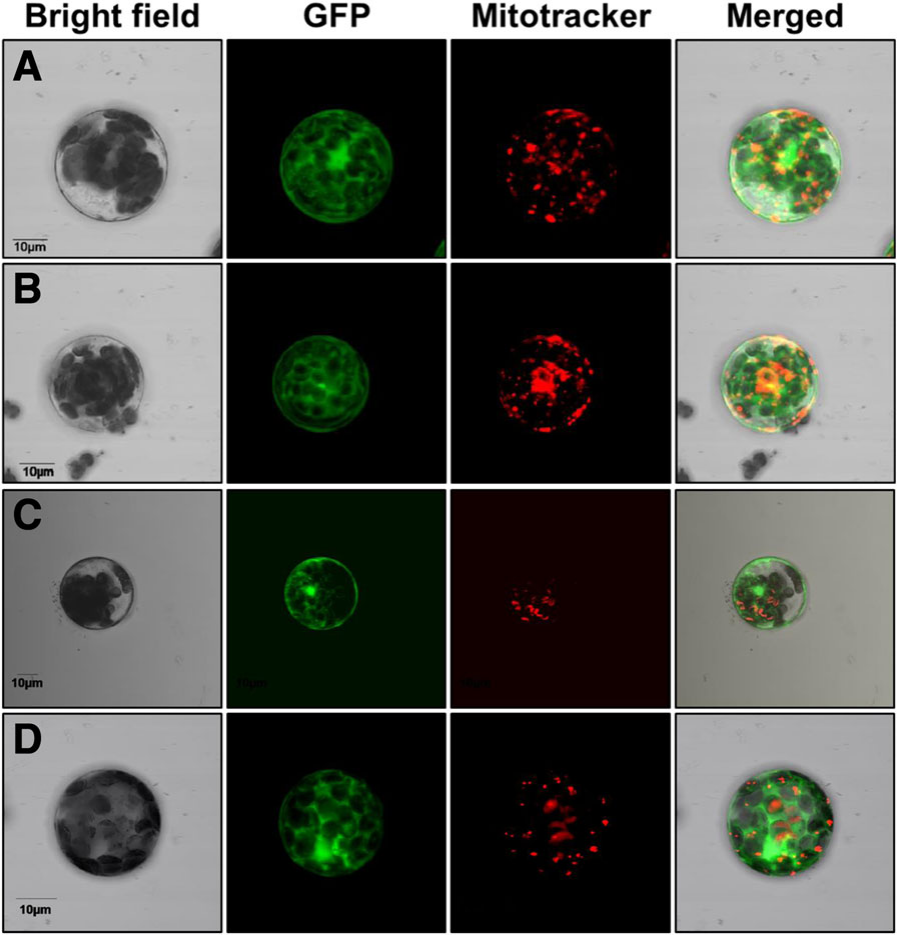
A functional mitochondrial anchor peptide is included in the first 30 amino acids of maize HXKs. Zeta-projection images of protoplasts from *A. thaliana* transformed with the truncated versions (**a**) ZmHXK4Δ30, (**b**) ZmHXK5Δ30, (**c**) ZmHXK6Δ30 and (**d**) ZmHXK9Δ30, translationally fused at the GFP carboxy-terminus, are shown. GFP signal shown in green, Mitotracker was used as mitochondrial marker, and it is shown in red. The merge corresponds to signals of bright field, GFP and Mitotracker channels or bright field, GFP and chlorophyll channels. Bar = 10 μm. Representative images of four independent replicates

The mitochondrial localization could be mediated by an anchor peptide present at the amino-terminal sequence. Taking advantage of having full and truncated versions of these HXKs, it was possible to investigate the function of this putative membrane anchor peptide. As ZmHXK4Δ30:GFP, ZmHXK5Δ30:GFP, ZmHXK6Δ30:GFP, and ZmHXK9Δ30:GFP changed their localization from the mitochondria to the cytosol, we concluded that their 30 N-terminal amino acids represent functional mitochondrial anchor sequences to the mitochondria membrane (Fig. 7a-d).

### HXK activity in different maize tissues

**Fig. 8:**
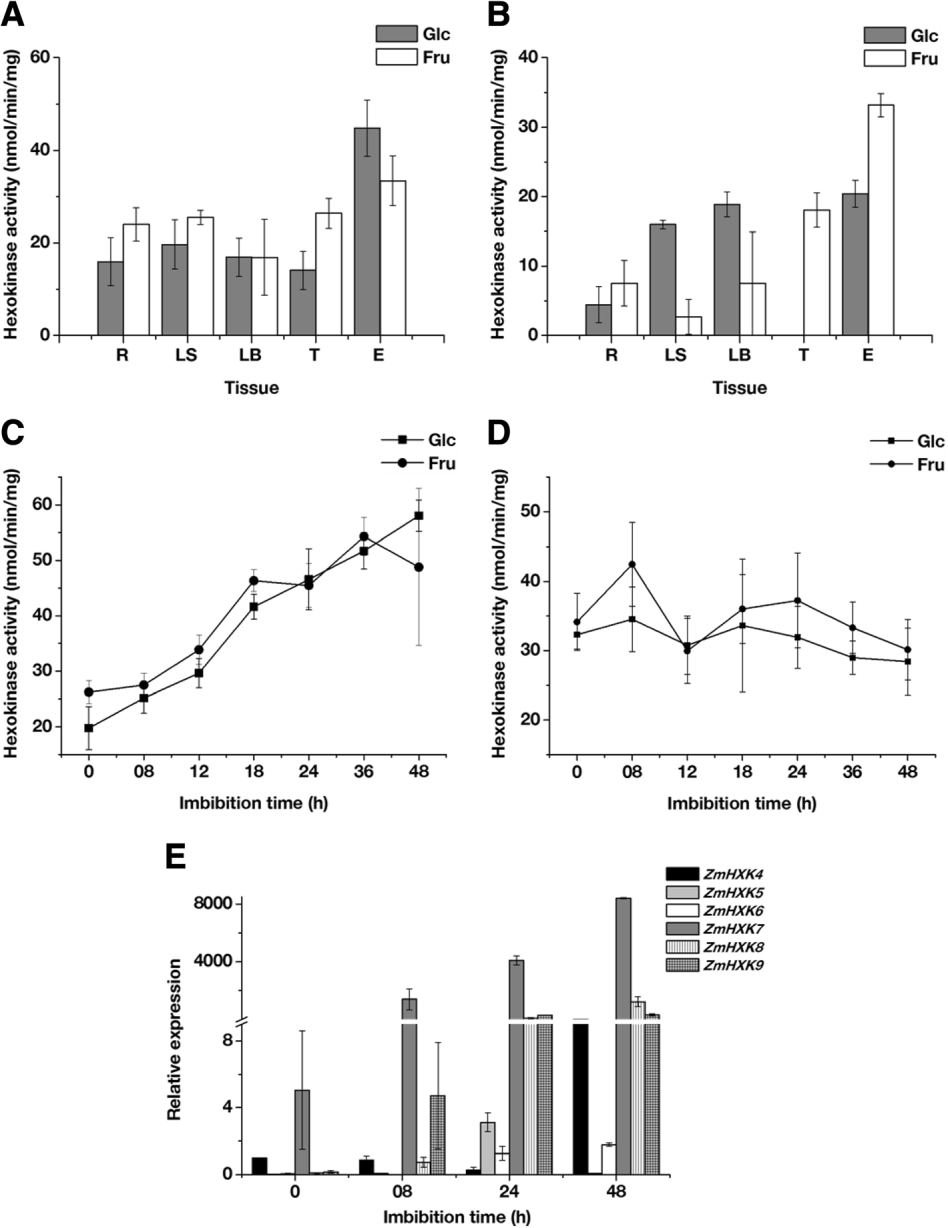
HXK activity and expression profiles of maize HXK genes during germination. HXK activities from mature plant roots (R), leaf blade (LB), leaf sheath (LS) and tassel (T), together with maize 24 h embryos (E) were determined using Glc or Fru as a substrate in (**a**) Cytosolic and (**b**) Mitochondrial fractions. HXK activities during maize embryo germination were determined using Glc or Fru as a substrate in (**c**) Cytosolic and (**d**) Mitochondrial fractions. **e** Relative expression of ZmHXK4–9 during embryo germination. The expression analysis was made by qPCR. The mean and standard deviations were obtained from three technical repeats with three independent replicates

As a first approximation to analyze the contribution of the HXKs to metabolism, we explored the HXK activity in different tissues of a mature plant and 24 h imbibed embryos. The embryo was chosen instead of the seed due to the low contribution of the endosperm storage reserves at the onset of the germination, and that the removal of the endosperm does not affect the germination [1, 4]. Cytosolic and mitochondrial fractions were obtained, and the quality of each fraction was assayed using western blotting. The cytosolic fraction was free of mitochondrial contamination, and the mitochondrial fraction was free of cytosolic fraction, however both fractions are contaminated with nuclei, and the mitochondria fraction also with microsomal fraction (Additional file 1: Figure S6). The phosphorylating activity was determined using both cytosolic and mitochondrial fractions with Glc and Fru as substrates (Fig. 8a and b). Glc and Fru phosphorylating activity of the cytosolic fraction was similar at the tissues of the mature plant, with higher activity in the 24 h imbibed embryos. The mitochondrial fractions from aerial tissues, leaf sheath and leaf blade, together with the embryo showed similar Glc phosphorylating activity and higher than the roots and tassel. The tassel and the embryo mitochondrial fraction have the highest Fru phosphorylating activity.

### HXK activity and expression profiles of maize HXKs during germination

Several molecules, including enzymes and mRNA that actively participate at the seed embryogenesis, are conserved and contribute to metabolism during germination [1, 2, 5]. Since high HXK activity was found in 24 h imbibed embryos, we analyzed the HXKs activity and the expression profile during the germination process (Fig. 8c-e). HXK activity was detected in the cytosol from dry embryos and increased until 48 h after imbibition (Fig. 8c). In contrast, the HXK activity at the mitochondrial fraction was similar to dry embryos at 48 h of imbibition (Fig. 8d). The contribution of the HXK activity of the cytosolic fraction at 48 h of embryo imbibition was higher than in the mitochondrial fraction (Fig. 8c-d). No differences in the phosphorylating activity were found using Glc or Fru as substrates (Fig. 8c-d).

The expression profile showed that the six analyzed maize HXKs were expressed during germination. However, each gene showed a particular expression profile (Fig. 8e). The mRNAs for *ZmHXK4* and *ZmHXK7* were detected in dry maize embryos, so they could be important during germination since they remain after the desiccation phase of seed maturation. Except at 48 h, *ZmHXK4* decreased with the germination time. The expression of *ZmHXK7, ZmHXK8* and *ZmHXK9* constantly increased during germination*,* with *ZmHXK7* showing the highest expression (Fig. 8e). At 24 h *ZmHXK6*, *ZmHXK7*, *ZmHXK8* and *ZmHXK9* are highly expressed, while low levels of expression were found for *ZmHXK4* and *ZmHXK5*.

## Discussion

In this study, we demonstrated that six maize HXKs, ZmHXK4–9, are functional. The ZmHXKs differ in the activity level and substrate specificity. ZmHXK9 has minimal activity compared to the others. ZmHXK4–8 phosphorylate Glc and Man with high affinity, with ZmHXK8 having the lower catalytic efficiency for Glu, Man and Fru than ZmHXK4–7. ZmHXK7 was the member with the highest efficiency constant for ATP. In *Arabidopsis*, from the three catalytic AtHXKs, only AtHXK1 and AtHXK3 have been characterized enzymatically. AtHXK1 (mitochondrial attached protein) displays a *K*_m_ for Glc 89 μM, *K*_m_ Fru 17 mM and *K*_m_ ATP 9.4 μM. AtHXK3 (chloroplast protein) showed a *K*_mapp_ Glc 61 μM, *K*_mapp_ Man 85 μM and *K*_mapp_ Fru 17 mM [18, 34, 41]. In rice, two of the ten members of the HXK family were characterized. OsHXK5 displayed a *K*_m_ for Glc 190 μM, and OsHXK6 has a *K*_m_ for Glc 200 μM [23]. The *K*_m_ values for the hexoses in rice and *Arabidopsis* are similar with those obtained in this work. Moreover, five maize HXKs showed low affinity for Fru similar to HXKs from yeast, rat, parasites, pea seeds, potato tubers and spinach [7, 42, 43]. Unlike fructokinases, in vivo HXKs were suggested to preferably phosphorylate Glc rather than Fru because the Fru *K*_m_ values were 8.7–22 mM for HXK and 0.001–0.10 mM for fructokinases [10, 44, 45].

An interesting feature of a group of maize HXKs is that the 30 amino acids at the N-terminus make the proteins insoluble in *E. coli* and for some of them also modify their activity in the yeast mutant. There are several possible explanations for this effect. First, ZmHXK4–6 and 9 may be inserted to the outer membrane of the mitochondria, a feature that could be relevant since the three hexose-phosphorylating enzymes are cytosolic in the wild-type yeast strain [46]. Therefore, a different subcellular localization of HXKs could affect substrate availability. Second, the complete version of the enzyme possibly aggregates in the cytosol and reduces its activity. Third, the HXK N-terminus could be embedded in yeast membranes in a different way than in the plant mitochondrial membrane, resulting in altered enzyme activity. Thus far, these or other possibilities have not been considered and remain to be understood.

Conversely, the elimination of 30 amino acids at the N-terminus of ZmHXK9 demonstrated that this protein has enzymatic activity and is sufficient to allow for yeast growth in a medium supplemented with Glc or Fru as the carbon source. Therefore, ZmHXK4–9 must be functional HXKs, even if ZmHXK9 shows a marginal enzymatic activity when is produced in *E. coli*, then its physiological relevance remains uncertain. The low activity of ZmHXK9 could be due to the cooperative effect of amino acid changes, like the presence of many synonymous amino acid substitutions in the hydrophobic channel of ZmHXK9 (see Additional file 1: Table S2 and Figure S7) that could affect enzyme activity, even if the hydrophobicity of the putative proton channel is retained; 2) ZmHXK9 has six additional polar amino acids at the large domain near the hinge (Additional file 1: Figure S7). A longer domain could affect the flexibility of the hinge and reduce interdomain interactions between the substrates and the large and small domains; 3) the change of Pro^133^ → Glu^133^ (position relative to AtHXK1) and Gly^310^ → Asp^310^ (see Additional file 1: Figure S7), which could increase the enzyme flexibility and affects Glc binding. Gly 310 is conserved in catalytic enzymes but it is absent in HKL—such as AtHXL1, AtHXL2, OsHXK3, and OsHXK10—and in the predicted ZmHXK3a, ZmHXK3b, and ZmHXK10 [20, 25, 32, 47].

The metabolic responses to the substrate and product concentration combined with their subcellular localization might contribute to HXK activity and function. One reported classification recognized four types of HXKs in plants. We were able to differentiate between two types, cytosolic and mitochondrial HXK isoenzymes, using two methods. The first was to determine the effect of ADP, NAG and G6P. This allowed for us to differentiate two types of maize HXKs, one with high sensitivity to these molecules such as ZmHXK4–6, and the other with low sensitivity such as ZmHXK7–8. There is no information about the sensitivities of the enzymes in rice or *Arabidopsis* for any inhibitor [9]. However, previous results using cytosolic and mitochondrial maize root fractions are similar to what we found in this study [21, 27, 34]. The sensitivity to inhibitors together with the localization studies using the full and truncated version of each HXK confirmed that in maize, there are apparently only two types of HXKs: B and C. Type B HXKs have a membrane anchor amino acid sequence that directs the protein to the mitochondria (Fig. 2b, Additional file 1: Table S3). In maize, the type B HXK members are ZmHXK4, ZmHXK5, ZmHXK6, and ZmHXK9. Conversely, type C or cytosolic HXKs are present only in monocotyledonous plants and the moss *Physcomitrella patens* [9, 12, 25, 48]. In maize, they are represented by ZmHXK7 and ZmHXK8.

There are no chloroplast or Golgi HXKs as suggested before [9, 26], the localization analysis on protoplast corroborates the phylogenetic analysis, in which only dicot plants have plastid HXK (Additional file 1: Figure S1). The absence of a chloroplast HXK (type A HXKs) in maize suggests that Glc is phosphorylated by cytosolic isoenzymes and used in starch and fatty acid synthesis when night-time energy supply is limited in the chloroplast [24, 37, 48–51]. However, it was also suggested that type A HXKs are only expressed in plants that tend to accumulate starch, which is not the case in maize [25, 52]. Either ZmHXK7 or ZmHXK8 could participate in these processes.

The first 30 amino acids at the N-terminus in ZmHXK4, ZmHXK5, and ZmHXK9 are necessary and sufficient to attach these proteins to the mitochondria in addition to their role in modifying the enzymatic activity (Fig. 4). In maize, one HXK was solubilized from the mitochondria using the zwitterionic detergent CHAPS, demonstrating their integration into the outer mitochondrial membrane. Apparently, the large hydrophobic peptide at the N-terminus from plant HXKs is inserted in the outer membrane of the mitochondria in a manner different than mammalian HXKs. This difference was probably the reason for the high resistance to G6P to the clotrimazole drug inhibition shown by plant HXKs as G6P and clotrimazole induce HXK detachment from the mitochondria in mammals [28]. The role of the first 30 amino acids in ZmHXK6 is not clear because the low level of cytosolic protein that was found was accompanied by large fluorescence aggregates (Fig. 4c, Fig. 5c). This fluorescence pattern was also observed in another type B HXK [36–38].

HXKs attached to the outer mitochondrial membrane are involved in numerous physiological processes given their strategic localization. This was suggested to represent an advantage over the use of ATP. For instance, in *A. thaliana,* HXKs are embedded in the outer mitochondrial membrane as a part of the glycolytic metabolon [39, 40]. In potato, mitochondrial HXKs regulate ROS in tissues with an intensive oxidative metabolism [34]. In tobacco, mitochondrial HXK protects against programmed cell death induced by ROS and confers plant resistance to pathogen attack [53, 54]. We speculate each one might play a different role in the cell that due to the variability of Type B HXKs in maize.

During germination, the quiescent dry seed resumes its metabolic activity with water uptake, and the carbon source from embryonic tissues is required for completing the germination [2, 5]. The transcripts for mitochondrial HXKs *ZmHXK5* and *ZmHXK6* were detected at low levels in dry maize embryos (Fig. 8e). They may participate in seed embryogenesis because they remain after the desiccation phase. However, at early stages of germination, the mitochondrial HXK activity was high, and it was maintained until 48 h of germination (Fig. 8d), even though there is an increase in the oxygen uptake with the consequent stimulation of mitochondrial biogenesis [4, 5, 55]. The activity of mitochondrial HXKs like ZmHXK4 that was highly expressed in embryos after 8 h of germination could be important to the general metabolism. The high expression of *ZmHXK9* is intriguing since very low activity was detected for this enzyme, but its participation together with ZmHXK4–6 cannot be excluded. This could indicate a metabolic and a sensor role. Furthermore, since the half maximal inhibitory concentration (IC_50_ values) of the mitochondrial isoforms is similar (Table 2), it would be difficult to use these inhibitors to distinguish their effects.

In regard to the cytosolic HXKs, during the embryo development, two cytosolic HXKs show a considerable difference in their Km for Glc [17, 56]. According to the kinetic characteristics and cytosolic localization of the recombinant enzymes analyzed here, the described activity of high- and low-affinity enzymes could be attributed to ZmHXK7 and ZmHXK8, respectively. During maize seed development, increased HXK activity coincides with the active phase of fatty acid synthesis. ZmHXK7 and ZmHXK8 could supply G6P to feed both the OPPP and fatty acid synthesis in the plastid to store reserves in the scutellum [57, 58]. However, the metabolic role during germination could be different since different metabolites are present. In rape, cauliflower, sugar beet, and radish, the content of ATP ranged from 0 to 0.2 nmol/seed in non-imbibed seeds and rise to 0.3 to 1.2 after 3 h of imbibition [59, 60]. The high expression of *ZmHXK7*, an enzyme with a small *K*_*m*_ for ATP and low sensitivity for ADP, may confer an advantage for the cell at early times in germination to start the hexose metabolism. In rice, the orthologous gene *OsHXK7* is necessary to complete germination under hypoxia, suggesting an important role in ATP generation mainly through fermentation [12, 61]. This result might be explained by the biochemical characteristics of the protein identified in this study and the presence of the *ZmHXK7* transcripts.

In germinated embryos, the reduction in lipid reserves in the scutellum overlaps with the sugar biosynthesis [5]. The transitory accumulation of sugars could be the driving force to express different set of HXK isoenzymes. For instance, the two cytosolic enzymes, ZmHXK7 and ZmHXK8, differ in two orders of magnitude in their specificity constant. The sudden increase in the *ZmHXK8* mRNA at 8 h of germination and the accompanied increase in the cytosolic HXK activity (Fig. 8c and e) could be associated with the required production of G6P to feed the OPPP, involved in cell wall and nucleic acids biosynthesis but also to create the appropriate redox state to promote the production of ATP through the electron transport chain and the reduction of disulfide bonds of important enzymes that promote the proteolysis and starch degradation to accelerate the reserve mobilization [1, 5].

## Conclusions

In this study, six HXKs from maize were characterized based on their substrate preferences, sensitivity to product inhibition and subcellular localization. We demonstrated that ZmHXK4–9 are functional HXKs. ZmHXK7 and ZmHXK8 are cytosolic proteins that phosphorylate Man and Glc, rather than Fru. Both enzymes have low sensitivity to inhibition by G6P and ADP with differences in their *K*_m_ value for Glc. By contrast, ZmHXK4–6 are mitochondrial proteins that phosphorylate Man, Glc, and Fru with a similar *K*_m_ for each hexose. Marginal HXK activity was found for ZmHXK9. At the N-terminus, in the ZmHXK4–6 and ZmHXK9 sequences, 30 amino acids were required to attach these proteins to the mitochondrial membrane, and their presence modified the HXK activity. We also show that dry and imbibed embryos express different sets of HXKs. ZmHXK4 and 7 transcripts were detected in dry embryos, while at 24 h of germination; the most abundant transcripts were ZmHXK5-9. Our results suggest that ZmHXK4 harbors the higher HXK activity detected in mitochondria. Likewise, ZmHXK7 contributes to the hexose phosphorylation at early germination times at the cytosol and together with ZmHXK8 at later times, considering the differences in their biochemical parameters we suggest that they may not only be redundant proteins but may also have specific roles depending on carbon availability, metabolic needs, or sensor requirements. Further investigation is required to understand their specific physiological roles as sensors or metabolite producers during various developmental processes using transgenic plants with gene knock outs.

## Methods

### Plant materials

*Zea mays* var. Chalqueño seed embryos were obtained by manual dissection. Embryos were disinfected with a 0.12% hypochlorite solution and allowed to grow in 1% agar in different times at 29 °C in darkness. Mature maize plants were obtained by planting seeds in an experimental field close to Facultad de Química, UNAM (19°19′24.4”N 99°10′37.9”W) and the tissues were collected after 3 months of growth. Maize tissues were frozen in liquid nitrogen, ground to a fine powder, and stored at − 80 °C until use. All maize tissues were used to determine the HXK activity. Embryos were also used to determine the expression profile of ZmHXKs along the embryo germination and to clone the ORF of six putative catalytically active HXKs. The HXK activity of each recombinant protein was determined in vitro and by yeast complementation assay. The subcellular localization of ZmHXK4–9 was made in protoplast from *Arabidopsis thaliana* plants. The *A. thaliana* seeds ecotype Columbia (Col-0) were obtained from ABRC and were sown in pots containing Sunshine 3. After 2 days at 4 °C for stratification, plants were grown for 3–4 weeks in a growth chamber (120–150 μmol/m^2^sec, 20–22 °C).

### Analysis and subcellular localization prediction of maize HXKs

The gene sequences of maize, *A. thaliana* and rice HXKs were obtained from EnsemblPlants and NCBI. The ID of the genes under study is included in the Additional file 1: Table S1 . Sequence identity analyses were performed for ZmHXK3–10 with Clustal Omega (http://www.ebi.ac.uk/Tools/msa/clustalo/). SeaView 4 software was used to search for conserved domains by inspection [33] using sites that are present in AtHXK1 as a reference [20, 33]. TargetP 1.1 and Toppred 1.10 were used to predict subcellular protein localization http://www.cbs.dtu.dk/services/TargetP/) [35]*,* and http://mobyle.pasteur.fr/cgi-bin/portal.py#forms::toppred.

### RT-qPCR analysis

Maize embryo total RNA was extracted using TRIzol™ Reagent (Invitrogen™, Thermo Fisher Scientific) according the manufacturer’s instructions. RNA purity and integrity were assessed on an agarose gel using the 28S and 18S ribosomal band’s intensity as reference. One microgram of total RNA was used for reverse transcription with the ImProm-II™ Reverse Transcription System (Promega). Relative expression of *ZmHXK4–9* in the embryo tissues was obtained by qPCR. To ensure the validity of the results we evaluated the specificity and efficiency of the qPCR for each pair of primers used to amplify the HXK (Additional file 1: Table S4). The set of primers with efficiency above 95% were chosen. The efficiency was calculated using a calibration curve with a serial dilution of cDNA (1,1; 1:10; 1:100; 1:1000; 1:10000) and the formula: E = (10^[− 1/m]^)*100, where m is the slope curve of Ct vs logarithmic concentration. qPCR was performed in the thermocycler 7500 Real-Time PCR System (Applied Biosystems). The PCR Reaction mix contained 10 μL SYBR Green Master Mix (Applied Biosystems), 0.15 μL primer forward 20 μM, 0.15 μL primer reverse 20 μM, 2 μL cDNA, and 7.7 μL nuclease free water. Expression of endogenous gene *Zm18s* [62] was used as control. Relative expression was calculated using the formula: Ratio expression = E_target_^(ΔCPtarget(control-sample)^/E_ref_^(ΔCPref(control-sample)^ [63], where E_target_ is the amplification efficiency of the target gene, ΔCP_target_ is the Ct value of the control sample (*ZmHXK4* expression at 0 h) minus Ct value of treated samples, E_ref_ is the amplification efficiency of the endogenous gene, and ΔCP_ref_ is the Ct value of endogenous gene in the control sample minus Ct value of endogenous gene in treatment samples [63].

The PCR conditions were as follows: one cycle of 3 min at 94 °C, the amplification was done for 30 s at 98 °C, 1 min at 60 °C, 1 min at 72 °C for 28 cycles for *ZmHXK7* and *ZmHXK8* and for *ZmHXK4-ZmHXK6* and *ZmHXK9* was 30 cycles, and finally, one cycle of 10 min at 72 °C. PCR products were resolved by electrophoresis on 2.0% agarose gels. Each experiment was executed from the same quantity of total RNA.

### Cloning and plasmid constructs

Maize HXK clones were obtained from cDNA of 24 h imbibed embryos. Primers were designed for the candidate genes specific 5′ and 3′ untranslated regions (UTRs) using the Primer3Plus software (http://www.bioinformatics.nl/cgi-bin/primer3plus/primer3plus.cgi): *ZmHXK4-UTR 5’-GGAGGGAGATTGGTTCGT-3*′ and *5’-CGGATAGCCACIDOCAAGGA-3′*, *ZmHXK5-UTR 5’-TAGTTCGCTGGAGGAGTTGG-3′* and *5’-TCGATCCACACCAGTTTTCA-3′*, *ZmHXK6-UTR 5’-AGCTGGTGCGTACTTGGTTT-3′* and *5’-GGATGGACGGCTTATTCA-3′*, *ZmHXK7-UTR 5′- GTCGCAGCTTTCTTTCGTGT-3′* and *5’-AGGATTCAGCACGAGTTGC-3′*, *ZmHXK8-UTR 5′- GCCAGGACTCTTGATTTGCT-3′* and *5’-CTGCAGACGCTGGACAGTTA-3′*, *ZmHXK9-UTR 5’-ACTCCCGTGGCAGTGATAC-3′* and *5’-CGTCGGATACACACAGAGG-3′*. The *AtHXK1* clone was obtained from the cDNA of 7-day-old *A. thaliana* seedlings; the primer pair used was also designed with Primer3Plus software (*AtHXK1-UTR 5’-ATGGGTAAAGTAGCTGTTGGAG-3′* and *5’TTAAGAGTCTTCAAGGTAGAGAGAGTG-3′*). The PCR conditions were the same as described above. PCR products were resolved by electrophoresis on 2.0% agarose gels. PCR products were purified from preparative gels and cloned in the pGEM®-T Easy vector (Promega) with T4 DNA ligase according to the manufacturer’s instructions. Ligation products were used to transform the competent *Escherichia coli* DH5α. The recombinant vectors were sequenced to verify the clone identity and insert direction. Gateway® technology was used to subclone the different HXKs into the donor and destination vectors. The ORFs of the HXKs genes that lacked the stop codon were flanked with *attB/P* sequences through two PCR rounds employing pGEM-HXK constructs (Additional file 1: Table S4) as template and using the Advantage HD polymerase (Clontech). The products were cloned into the pDNOR™221 vector (Thermo Fisher Scientific) by BP clonase (Invitrogen) to generate entry clones. Plasmid constructs in the pDNOR™221 vector encoding N-terminal truncated variants of *ZmHXK4, ZmHXK5, ZmHXK6* and *ZmHXK9* were produced by amplifying the ORF of each HXK gene that lacked the 87 nucleotides after the start codon (Additional file 1: Table S4). These variants were named *ZmHXK4Δ30, ZmHXK5Δ30, ZmHXK6Δ30* and *ZmHXK9Δ30*. The obtained constructs were sequenced to confirm deletion and correct insertion of the gene into the vector.

### Expression and purification of recombinant maize HXKs

HXK subcloning from the entry clone to destination vector pET-DEST™42 (Thermo Fisher Scientific) was performed with LR clonase (Invitrogen) according to the manufacturer’s instructions. This vector added a V5 epitope and 6xHis tag at the carboxy-terminus of each HXK for detection and protein purification. BL21-CodonPlus™ (DE3)-RIL (Stratagene) competent *E. coli* cells were transformed with the expression clones. For protein induction, 100 μL of each clone was grown in 100 mL of LB supplemented with 100 μg/mL ampicillin and 34 μg/mL chloramphenicol and incubated for 16 h at 37 °C under constant agitation (200 rpm). The saturated culture was added to 1 L of LB and incubated at 37 °C with constant agitation (150 rpm). Cells were induced at 0.600 units of absorbance at 600 ηm with 0.75 mM Isopropyl β-D-1-thiogalactopyranoside (Promega) and grown at 25 °C for 2 h for ZmHXK7 and ZmHXK8; 4 h for ZmHXK4Δ30, ZmHXK5Δ30 and ZmHXK9Δ30; and 6 h for ZmHXK6Δ30. Cells were harvested by centrifugation (5 min at 4000 *xg*), and the pellet was resuspended in 30 mL of lysis buffer (500 mM NaCl, 50 mM NaH_2_PO_4_/Na_2_HPO_4_ pH 8.0 and 5 mM benzamidine) and incubated on ice for 10 min. The cell suspension was sonicated for 5 min with 15 s pulses at intervals of 15 s. After 5 min of centrifugation at 13,000 *xg* at 4 °C, the supernatant was loaded onto a Ni^2+^ affinity column (05893682001, Roche) previously equilibrated with 30 mL of equilibrium buffer (50 mM NaH_2_PO_4_/ Na_2_HPO_4_ pH 8.0, 300 mM NaCl, 20 mM Imidazol and 5 mM benzamidine) and incubated for 2 h at 4 °C. The column was washed with 60 mL of equilibrium buffer, and the bound protein was eluted with 6 mL of elution buffer (50 mM pH 8.0 NaH_2_PO_4_/Na_2_HPO_4_, 300 mM NaCl, 5 mM benzamidine and Imidazol 50–250 mM) and collected in 1 mL fractions. All the fractions were concentrated by centrifugation, and the buffer was exchanged for 50 mM NaH_2_PO_4_/Na_2_HPO_4_ pH 8.0 and 5 mM benzamidine, with an Amicon 30 kDa (Millipore). Glycerol was added to each concentrated fraction to a 10% final concentration and stored at − 20 °C until use.

### Recombinant protein detection

Purified recombinant HXK fractions were separated in a 12% SDS-PAGE and transferred in a wet chamber to a polyviniledene fluoride membrane (PVDF) according to Tobwin et al., [64]. The membranes were blocked for 1 h with 5% defatted milk in TTBS (20 mM Tris-HCl, pH 7.5, 0.1% Tween-20 and 150 mM NaCl). Since all the proteins have the V5 epitope at the C-terminal, the membranes were probed with a 1:5000 dilution of Anti-V5-HRP (R961–25, Invitrogen), and incubated for 1.5 h at 30 °C. To develop the peroxidase reaction chemiluminescent substrate was added (WBKLS0500, Merck-Millipore) and detected with the ChemiDoc™MP (Bio-Rad).

### Isolation of cytosolic and mitochondrial fractions

Roots, leaf blades, leaf sheaths and tassels of three months maize plants and the 24 h imbibed seed embryos were frozen in liquid N_2_ and kept at − 70 °C. Two grams of each tissue was crushed, and the fine powder was homogenized in buffer (50 mM Hepes/KOH pH 7.0, 300 mM sorbitol, 1 mM EDTA, 2 mM DTT, 1 mM PMSF and 1 tablet of protease inhibitor cocktail per 50 mL, Complete, Roche, Mannheim, Germany) in a ratio of 2 volumes per 1 g of tissue, using a tissue tearor homogenizer (Biospec Products, Daigger and Co. Inc. Vernon Hill, Il, USA) for 60 s. The tissue was filtered through a four-layer of pre-wet cheesecloth; the filtrate was centrifuged 5 min at 2400 g at 4 °C. The supernatant was centrifuged at 14,000 g for 10 min. The pellet contained the mitochondria fraction and the supernatant contained the cytosolic fraction. The supernatant was centrifuged at 110,000 g for 45 min in a TLA-100.4 rotor in an Optima TL-100 Centrifuge (Beckman, Palo Alto, CA, USA) at 4 °C to eliminate the microsomal fraction. The supernatant or cytosolic fraction was supplemented with 10% glycerol and stored at − 70 °C until use. The mitochondrial fraction was washed with 1 mL of homogenization buffer and centrifuged at 14,000 g for 10 min. The pellet was resuspended in 1 mL of homogenization buffer supplemented with 10% of glycerol and stored in aliquots at − 70 °C until use.

### Protein determination

The protein concentration was determined by the Bradford method [65] using BSA as the standard.

### HXK activity assay

HXK activity assays were performed as described previously by Dai [18] with some modifications. Briefly, the assay reaction was performed in a 96 microplate format to determine both Glc and Fru HXK activity. The reaction medium contained 50 mM NaH_2_PO_4_/Na_2_HPO_4_ pH 8.0, 2 mM MgCl_2_, 1 mM EDTA, 15 mM KCl, 2 mM ATP, 2 mM phosphoenolpyruvate, 1 U/mL Glc-6-phosphate dehydrogenase, 7 U/mL pyruvate kinase (PK) and 4 mM NADP^+^, 0.01–100 mM Glc or 0.5–200 mM Fru in a 250 μL final volume. When Fru was the substrate, 3 U/mL phosphoglucose isomerase was added. To obtain the ATP *K*_*m*_ value, its concentration was set in the range of 10 to 1000 μM with a fixed Glc concentration of 5 mM except for ZmHXK8 that was set at 50 mM. To HXK inhibition assays, ADP concentrations ranged from 0.5 to 15 mM and N-acetylglucosamine (NAG) from 1 to 150 mM with fixed 5 mM Glc and 2 mM ATP, except for ZmHXK8 in which 50 mM Glc was set. In these three cases, 5 or 50 mM Glc was used as a substrate. The reaction medium was different both to determine the Man HXK activity, and when Glc-6-P (G6P) was used as the HXK inhibitor, the reaction mixture contained 50 mM NaH_2_PO_4_/ Na_2_HPO_4_ pH 8.0, 2 mM MgCl_2_, 1 mM EDTA, 15 mM KCl, 2 mM ATP, 7 U/mL PK, 10 U/mL Lactate dehydrogenase and 2.5 mM NADH. Man was assayed from 0.5 to 100 mM and 10 to 150 mM for G6P inhibition. The reaction started by adding 2.5 μg of recombinant protein or 40 μg of mitochondrial or cytosolic fractions. The absorbance at 340 nm was read every 15 s for 5 min with an Epoch™ microplate reader (Bio Tek). The curves were analyzed with the Gen5™ Microplate Data Analysis software. The *K*_m_, *k*_cat_, *V*_max_ and IC_50_ values were obtained with OriginPro 8.0 software (OriginLab Corporation).

### Yeast complementation assay

Maize HXKs (full and truncated versions without any tag) and *AtHXK1* entry clones were subcloned into the pDRf1 vector [66] (Addgene plasmid # 36026) using the Gateway® technology. *S. cerevisiae* JT 20088 genetic background W303-1a (*hxk1::HIS3 hxk2::LEU2 glk1::LEU2*) was transformed with each pDRf1-HXK construct according to Gietz [67]. The results of the complementation assay were verified by screening transformants in a solid plate of SGal-URA (synthetic defined medium with galactose and uracil). Selected clones were grown overnight in SGal-URA liquid medium. The next day, 250 μL of saturated medium was added to 1 ml of fresh SGal-URA to reach an absorbance between 0.5–0.6 units at 600 nm. Tenfold serial dilution were made from this culture, and 10 μL of each dilution was grown in SGal-URA, SGlc-URA, SFru-URA plates and incubated at 30 °C for 2 days.

An expression analysis for each *ZmHXK* gene was made for each yeast mutant. The RNA was extracted using TRIzol™ Reagent (Invitrogen™, Thermo Fisher Scientific) according the manufacturer’s instructions. The cDNA was produced as described before and the list of primers used for this assay is in the Additional file 1: Table S4. The PCR conditions were as follows: one cycle of 3 min at 94 °C, the amplification was done for 30 s at 98 °C, 1 min at 60 °C, 1 min at 72 °C for 28 cycles for *ZmHXK7* and *ZmHXK8* and for *ZmHXK4-ZmHXK6* and *ZmHXK9* was 30 cycles, and finally, one cycle of 10 min at 72 °C. PCR products were resolved by electrophoresis on 2.0% agarose gels. Each experiment was executed from the same quantity of total RNA.

### Subcellular localization of ZmHXK:GFP

HXK entry clones were digested with the restriction enzyme *MluI* (FD0564, Thermo Scientific), and fragments of approximately 3000 bp were subcloned into the pEarleyGate 103 binary vector using the Gateway® technology [68]. To express the HXK-GFP fusions in a transient assay, *A. thaliana* protoplasts were made according to Yoo [69]. Leaves from 3 to 4 weeks old of *A. thaliana* (Col-0) plants were used for protoplast isolation. Transfection was performed via the PEG-calcium method. As a control of transfection, the plasmid HBT-sGFP (S65 T)-NOS was used (Stock # CD3–911 from ABRC/https://www.arabidopsis.org). After 18 h of transfection, protoplasts were stained to visualize the mitochondria with 75 ηM Mitotracker® RED CMXRos (Molecular Probes, Eugene, OR) for 5 min in dark conditions. GFP fluorescence was monitored with a confocal Microscope Olympus FV1000 using 488 nm/505–545 nm (excitation/emission) and 581 nm/644 nm (excitation/emission).

## Additional file

Additional file 1: **Information Figure S1 and Table S1**. **Figure S1**. Molecular phylogenetic analysis for plant HXKs. **Table S1**. ID numbers of the HXK gene families used for the phylogenetic analysis. **Table S2**. Comparison of conserved amino acids at catalytic and substrate binding domains between ZmHXKs with AtHXK1 [33]. **Figure S2**. SDS-PAGE of full versions of recombinant ZmHXKs. Purification process of recombinant: (A) ZmHXK4, (B) ZmHXK5, (C) ZmHXK6, (D) ZmHXK7, and (E) ZmHXK8. St: Molecular weight standard, P: Pellet clarified with urea, S: Soluble supernatant, U: Unbound, W: Wash, EX: Elution. **Figure S3**. Purification of truncated versions of recombinant ZmHXKs. Purification process of recombinant: (A) ZmHXK4Δ30, (B) ZmHXK5Δ30, (C) ZmHXK6Δ30 and (D) ZmHXK9Δ30. All fractions were separated by SDS-PAGE and stained with Coomasie blue. (E) Western Blot of full and truncated versions of ZmHXK4-6. The proteins were detected using the anti-V5-HRP. St: Molecular weight standard, T: Total cell extract S: Soluble supernatant, U: Unbound, W: Wash, EX: Elution. **Figure S4**. Inhibitory effects of ADP, NAG and G6P on ZmHXKs. (A, B, C) ZmHXKΔ4, (D, E, F) ZmHXKΔ5, (G, H, I) ZmHXKΔ6, (J, K, L) ZmHXK7, and (M, N, O) ZmHXK8. **Figure S5**. Expression profile of full and truncated versions of ZmHXKs in the JT 20088 yeast mutant. **Table S3**. Subcellular prediction of ZmHXKs.**Figure S6**. Evaluation of cytosolic and mitochondrial purity using specific antibodies. The purity of the cytosolic (Cyt), mitochondrial washed (wMit) and mitochondrial Percoll purified (pMit) fractions (10 µg) was evaluated by Western blot using Agrisera (Vännäs, Sweden) antibodies. These are representative membranes of at least three replicates. **Figure S7**. Changes in the amino acids of ZmHXK9 that could explain its low activity. The sequences were aligned using SeaView 4 [33]. **Table S4**. List of primers used for qPCR analysis, subcloning each maize HXK and PCR analysis in the yeast mutant. Uniprot^1^ (https://www.uniprot.org/uniprot The UniProt Consortium. UniProt: the universal protein knowledgebase. Nucleic Acids Res. 2017;45:D158–9), PLAZA^2^ (https://bioinformatics.psb.ugent.be/plaza/), EnsemblPlants^3^ (http://plants.ensembl.org/index.html) and NCBI4 (https://www.ncbi.nlm.nih.gov/). **Table S2** Comparison of conserved amino acids at catalytic and substrate binding domains between maize HXKs with AtHXK1 [33]. **Figure S2**: **(A)** ZmHXK4, **(B)** ZmHXK5, **(C)** (PDF 1360 kb)

## Abbreviations

Fru: Fructose
G6P: Glc-6-P
GFP: Green Fluorescent Protein
Glc: Glucose
HKL: HXK-like
HXK: Hexokinase
IC_50_: half maximal inhibitory
Man: D-Mannose
NAG: N-Acetylglucosamine
OPPP: oxidative pentose phosphate pathway, ORF, open reading frame (ORF)
PVDF: polyviniledene fluoride membrane
SFru-URA: Synthetic defined medium with Fru and uracil
SGal-URA: Synthetic defined medium with Galactose and uracil
SGlc-URA: Synthetic defined medium with Glu and uracil
TCA: tricarboxylic acids cycle
