# Supplementary material for: Biochemical properties and subcellular localization of six members of the HXK family in maize and its metabolic contribution to embryo germination
Source: BMC Plant Biol. 2019 Jan 15;19:27. doi: 10.1186/s12870-018-1605-x (PMC6332545; doi:10.1186/s12870-018-1605-x)
Supplement: Supplementary file 1 — Information Figure S1 and Table S1. Figure S1. Molecular phylogenetic analysis for plant HXKs. Table S1. ID numbers of the HXK gene families used for the phylogenetic analysis. Table S2. Comparison of conserved amino acids at catalytic and substrate binding domains between ZmHXKs with AtHXK1 [33]. Figure S2. SDS-PAGE of full versions of recombinant ZmHXKs. Purification process of recombinant: (A) ZmHXK4, (B) ZmHXK5, (C) ZmHXK6, (D) ZmHXK7, and (E) ZmHXK8. St: Molecular weight standard, P: Pellet clarified with urea, S: Soluble supernatant, U: Unbound, W: Wash, EX: Elution. Figure S3. Purification of truncated versions of recombinant ZmHXKs. Purification process of recombinant: (A) ZmHXK4Δ30, (B) ZmHXK5Δ30, (C) ZmHXK6Δ30 and (D) ZmHXK9Δ30. All fractions were separated by SDS-PAGE and stained with Coomasie blue. (E) Western Blot of full and truncated versions of ZmHXK4-6. The proteins were detected using the anti-V5-HRP. St: Molecular weight standard, T: Total cell extract S: Soluble supernatant, U: Unbound, W: Wash, EX: Elution. Figure S4. Inhibitory effects of ADP, NAG and G6P on ZmHXKs. (A, B, C) ZmHXKΔ4, (D, E, F) ZmHXKΔ5, (G, H, I) ZmHXKΔ6, (J, K, L) ZmHXK7, and (M, N, O) ZmHXK8. Figure S5. Expression profile of full and truncated versions of ZmHXKs in the JT 20088 yeast mutant. Table S3. Subcellular prediction of ZmHXKs.Figure S6. Evaluation of cytosolic and mitochondrial purity using specific antibodies. The purity of the cytosolic (Cyt), mitochondrial washed (wMit) and mitochondrial Percoll purified (pMit) fractions (10 µg) was evaluated by Western blot using Agrisera (Vännäs, Sweden) antibodies. These are representative membranes of at least three replicates. Figure S7. Changes in the amino acids of ZmHXK9 that could explain its low activity. The sequences were aligned using SeaView 4 [33]. Table S4. List of primers used for qPCR analysis, subcloning each maize HXK and PCR analysis in the yeast mutant. Uniprot1 (https://www.uniprot.org/uniprot The UniProt Con [file 12870_2018_1605_MOESM1_ESM.pdf]

**Information Fig S1 and Table S1.**

To represent the evolutionary relation among the plant HXKs presented in the Fig S1, the evolutionary history was inferred by using the Maximum Likelihood method based on the JTT matrix-based model using 2000 bootstrap [29, 30, 31]. The tree with the highest log likelihood (-40548.09) is shown. Initial tree(s) for the heuristic search were obtained automatically by applying Neighbor-Join and BioNJ algorithms to a matrix of pairwise distances estimated using a JTT model, and then selecting the topology with superior log likelihood value. The tree is drawn to scale, with branch lengths measured in the number of substitutions per site. The analysis involved 114 amino acid sequences. There was a total of 807 positions in the final dataset.

All amino acid sequences were aligned using MEGA X [29]. Group designations were made from visual inspection of apparent clusters and according to literature information. The amino acids sequences were obtained from Uniprot[1] (https://www.uniprot.org/uniprot The UniProt Consortium. UniProt: the universal protein knowledgebase. Nucleic Acids Res. 2017;45:D158-9), PLAZA[2] (https://bioinformatics.psb.ugent.be/plaza/), EnsemblPlants[3] (http://plants.ensembl.org/index.html) and NCBI[4] (https://www.ncbi.nlm.nih.gov/), for the following species:

*Sc: Saccharomyces cerevisiae; Mp: Marchantia polymorpha; Pp: Physcomitrella patens; Sm: Selaginella moellendorffii; Hv: Hordeum vulgare; Os: Oryza sativa ssp japonica; Sb: Sorghum bicolor; Zm: Zea mays; Ta: Triticum aestivum; At: Arabidopsis thaliana; Vv: Vitis vinifera; Sl: Solanum lycopersicum; St: Solanum tuberosum; Mt: Medicago truncatula; Nt: Nicotiana tabacum.* The gene and ID number of the analyzed sequences are in Table S1.

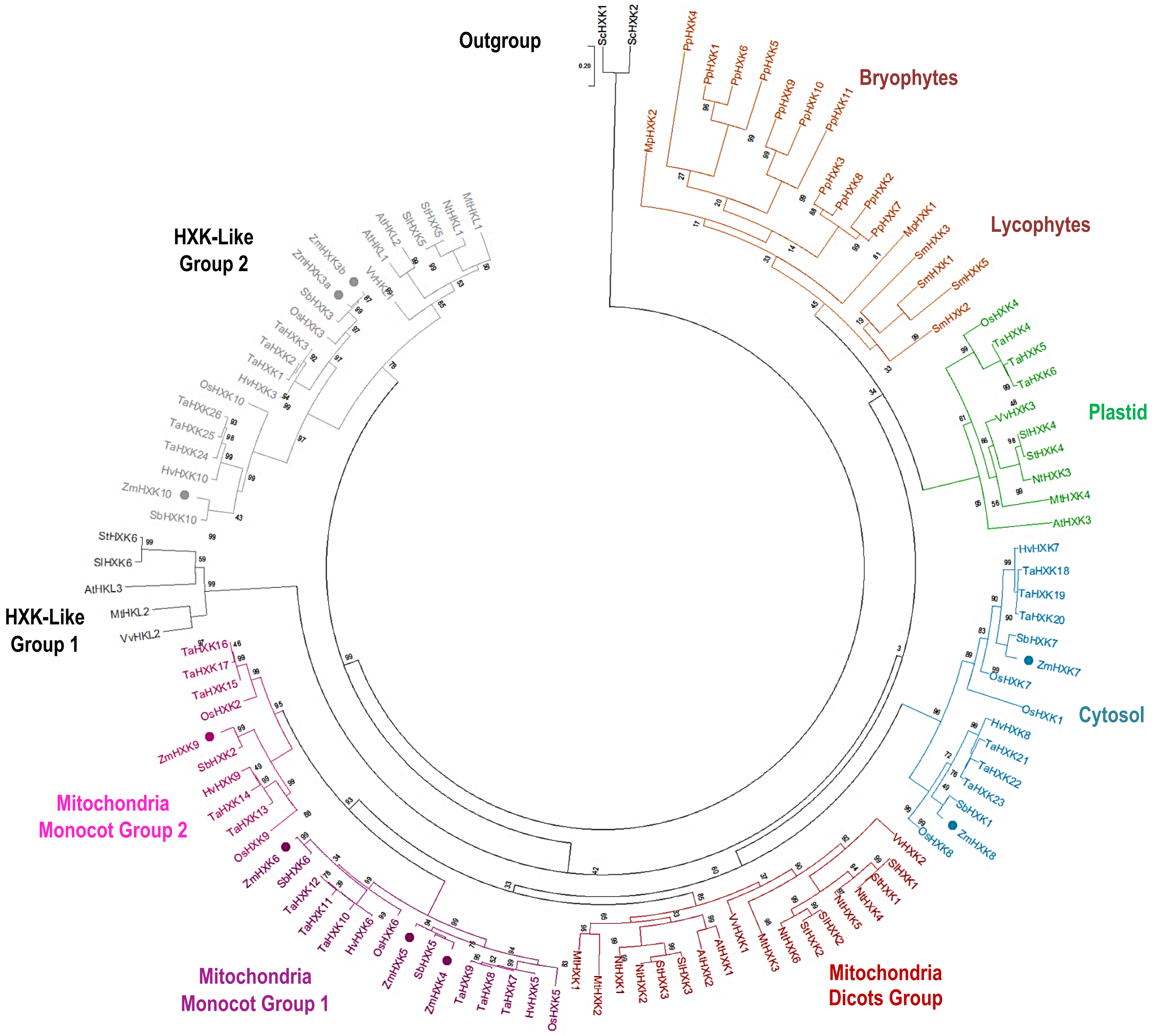


**Fig S1.** Molecular phylogenetic analysis for plant HXKs.

**Table S1.** ID numbers of the HXK gene families used for the phylogenetic analysis.

| Gene Name | ID number | Gene Name | ID number |
|---|---|---|---|
| ***Saccharomyces cerevisiae*** | | | |
| *ScHXK1* | P04806[1] | *ScHXK2* | P04807[1] |
| ***Marchantia polymorpha*** | | | |
| *MpHXK1* | Mapoly0022s0069[2] | *MpHXK2* | Mapoly0056s0008[1] |
| ***Physcomitrella patens*** | | | |
| *PpHXK1* | Pp3c14_6150[2] | *PpHXK2* | Pp3c19_20120[2] |
| *PpHXK3* | Pp3c21_19280[2] | *PpHXK4* | Pp3c1_5000[2] |
| *PpHXK5* | Pp3c2_11350[2] | *PpHXK6* | Pp3c10_8650[2] |
| *PpHXK7* | Pp3c22_9450[2] | *PpHXK8* | Pp3c18_12510[2] |
| *PpHXK9* | Pp3c8_18980[2] | *PpHXK10* | Pp3c23_970[2] |
| *PpHXK11* | Pp3c15_12840[2] | | |
| ***Selaginella moellendorffii*** | | | |
| *SmHXK1* | SMO134G0275[2] | *SmHXK2* | SMO147G0324[2] |
| *SmHXK3* | SMO141G0303[2] | *SmHXK5* | SMO230G0110[2] |
| ***Hordeum vulgare*** | | | |
| *HvHXK3* | HVU0038G3254[2] | *HvHXK5* | HVU0036G1776[2] |
| *HvHXK6* | HVU0038G0500[2] | *HvHXK7* | HVU0036G2577[2] |
| *HvHXK8* | HVU0038G2059[2] | *HvHXK9* | HVU0038G2827[2] |
| *HvHXK10* | HVU0036G1944[2] | | |
| ***Oryza sativa ssp japonica*** | | | |
| *OsHXK1* | OS07G0446800[3] | *OsHXK2* | XP_015637797[4] |
| *OsHXK3* | XP_015621344[4] | *OsHXK4* | XP_015645316[4] |
| *OsHXK5* | OS05G0522500[3] | *OsHXK6* | OS01G0742500[3] |
| *OsHXK7* | OS05G0187100[3] | *OsHXK8* | OS01G0190400[3] |
| *OsHXK9* | XP_015614778[4] | *OsHXK10* | OS05G0375100[3] |
| ***Sorghum bicolor*** | | | |
| *SbHXK1* | Sobic.003G035500[2] | *SbHXK2* | Sobic.003G280400[2] |

| | | | |
|---|---|---|---|
| *SbHXK3* | Sobic.003G421201[2] | *SbHXK5* | Sobic.009G203500[2] |
| *SbHXK6* | Sobic.003G291800[2] | *SbHXK7* | Sobic.009G069800[2] |
| *SbHXK10* | Sobic.009G119100[2] | | |
| ***Zea mays*** | | | |
| *ZmHXK3a* | GRMZM2G068913[3] | *ZmHXK3b* | GRMZM2G467069[3] |
| *ZmHXK4* | GRMZM2G058745[3] | *ZmHXK5* | GRMZM2G432801[3] |
| *ZmHXK6* | GRMZM5G856653[3] | *ZmHXK7* | GRMZM2G051806[3] |
| *ZmHXK8* | GRMZM2G104081[3] | *ZmHXK9* | GRMZM2G171373[3] |
| *ZmHXK10* | GRMZM2G046686[3] | | |
| ***Triticum aestivum*** | | | |
| *TaHXK1* | TAE02953G001[2] | *TaHXK2* | TAE54883G001[2] |
| *TaHXK3* | TAE52226G001[2] | *TaHXK4* | TAE05512G002[2] |
| *TaHXK5* | TAE56610G005[2] | *TaHXK6* | TAE57032G006[2] |
| *TaHXK7* | TAE37995G004[2] | *TaHXK8* | TAE47638G004[2] |
| *TaHXK9* | TAE40692G004[2] | *TaHXK10* | TAE57619G001[2] |
| *TaHXK11* | TAE13066G003[2] | *TaHXK12* | TAE40850G002[2] |
| *TaHXK13* | TAE40826G003[2] | *TaHXK14* | TAE19797G001[2] |
| *TaHXK15* | TAE41439G002[2] | *TaHXK16* | TAE21658G002[2] |
| *TaHXK17* | TAE05962G003[2] | *TaHXK18* | TAE02025G001[2] |
| *TaHXK19* | TAE30281G001[2] | *TaHXK20* | TAE51630G001[2] |
| *TaHXK21* | TAE08161G001[2] | *TaHXK22* | TAE51140G001[2] |
| *TaHXK23* | TAE53374G005[2] | *TaHXK24* | TAE08078G001[2] |
| *TaHXK25* | TAE49603G001[2] | *TaHXK26* | TAE51566G002[2] |
| ***Arabidopsis thaliana*** | | | |
| *AtHXK1* | AT4G29130[3] | *AtHXK2* | AT2G19860[3] |
| *AtHXK3* | AT1G47840[3] | *AtHKL1* | AT1G50460[3] |
| *AtHKL2* | AT3G20040[3] | *AHKL3* | AT4G37840[3] |
| ***Vitis vinifera*** | | | |
| *VvHXK1* | GSVIVG01015297001[2] | *VvHXK2* | GSVIVG01016971001[2] |
| *VvHXK3* | GSVIVG01009899001[2] | *VvHKL1* | GSVIVG01031551001[2] |

| | | | |
|---|---|---|---|
| *VvHKL2* | GSVIVG01002667001[2] | | |
| ***Solanum lycopersicum*** | | | |
| *SlHXK1* | Solyc03g121070.2[2] | *SlHXK2* | Solyc06g066440.2[2] |
| *SlHXK3* | Solyc12g008510.1[2] | *SlHXK4* | Solyc04g081400.2[2] |
| *SlHXK5* | Solyc11g065220.1[2] | *SlHXK6* | Solyc02g091830.2[2] |
| ***Solanum tuberosum*** | | | |
| *StHXK1* | PGSC0003DMG400002525[2] | *StHXK2* | PGSC0003DMG400016521[2] |
| *StHXK3* | PGSC0003DMG400000295[2] | *StHXK4* | PGSC0003DMG400009861[2] |
| *StHXK5* | PGSC0003DMG400013187[2] | *StHXK6* | PGSC0003DMG400030624[2] |
| ***Medicago truncatula*** | | | |
| *MtHXK1* | Medtr8g102460[2] | *MtHXK2* | Medtr6g088795[2] |
| *MtHXK3* | Medtr8g014530[2] | *MtHXK4* | Medtr1g025140[2] |
| *MtHKL1* | Medtr5g009000[2] | *MtHKL2* | Medtr4g097900[2] |
| ***Nicotiana tabacum*** | | | |
| *NtHXK1* | AAS60195[1] | *NtHXK2* | AAS60197[1] |
| *NtHXK3* | Q6Q8A5[1] | *NtHXK4* | AAT77515[1] |
| *NtHXK5* | AAS60192[1] | *NtHXK6* | AAS60194[1] |
| *NtHKL1* | AAS60198[1] | | |

**Table S2.** Comparison of conserved amino acids at catalytic and substrate binding domains between ZmHXKs with AtHXK1 [32]. The amino acid changes are in bold.

| Domain/ Residue | AtHXK1 | ZmHXK 3a | ZmHXK 3b | ZmHXK 4 | ZmHXK 5 | ZmHXK 6 | ZmHXK 7 | ZmHXK 8 | ZmHXK 9 | ZmHXK 10 |
|---|---|---|---|---|---|---|---|---|---|---|
| **Hydrophobic channel** | L 100 | **I 100** | **I 100** | L 109 | L 109 | L 109 | L 70 | L 72 | L 107 | **V 102** |
| | L 102 | L 102 | L 102 | L 111 | L 111 | L 111 | L 72 | L 74 | L 109 | L 104 |
| | L 143 | L 141 | L 141 | L 152 | L 152 | L 152 | L 113 | L 115 | L 150 | L 144 |
| | I 147 | **V 145** | **V 145** | I 156 | I 156 | I 156 | I 117 | I 119 | I 154 | I 148 |
| | L 151 | L 149 | L 149 | L 160 | L 160 | L 160 | L 121 | L 123 | L 158 | L 152 |
| | V 155 | V 153 | V 153 | V 164 | V 164 | V 164 | V 125 | V 127 | **I 162** | **I 156** |
| | L 172 | L 165 | L 165 | L 181 | L 181 | L 181 | L 138 | **I 144** | L 179 | L 168 |
| | F 174 | F 167 | F 167 | F 183 | F 183 | F 183 | F 140 | F 146 | F 181 | F 170 |
| | F 176 | F 169 | F 169 | F 185 | F 185 | F 185 | F 141 | F 148 | F 183 | F 172 |
| | F 197 | F 190 | F 190 | F 206 | F 206 | F 206 | F 163 | F 169 | F 204 | F 193 |
| | L 211 | L 204 | L 204 | L 220 | L 220 | L 220 | L 177 | L 183 | L 218 | L 207 |
| | L 215 | L 208 | L 208 | **M 224** | **M 224** | **M 224** | **M 181** | **M 187** | **I 222** | L 211 |
| **Catalytic residues** | K 195 | K 188 | K 188 | K 204 | K 204 | K 204 | K 161 | K 167 | K 202 | K 191 |
| | D 230 | D 223 | D 223 | D 239 | D 239 | D 239 | D 196 | D 202 | D 237 | **N 226** |
| **Glucose contacts** | T 194 | T 187 | **N 187** | T 203 | T 203 | T 203 | T 160 | T 166 | T 201 | T 190 |
| | K 195 | K 188 | K 188 | K 204 | K 204 | K 204 | K 161 | K 167 | K 202 | K 191 |
| | N 229 | N 222 | N 222 | N 238 | N 238 | N 238 | N 195 | N 201 | N 235 | N 225 |
| | D 230 | D 223 | D 223 | D 239 | D 239 | D 239 | D 196 | D 202 | D 236 | **N 226** |
| | S 177 | S 170 | S 170 | S 186 | S 186 | S 186 | S 143 | S 149 | S 184 | S 173 |
| | N 256 | N 249 | N 249 | N 265 | N 265 | N 265 | N 222 | N 228 | N 263 | N 252 |
| | E 284 | E 277 | E 277 | E 293 | E 293 | E 293 | E 253 | E 256 | E 291 | E 281 |
| | E 315 | E 308 | E 308 | E 324 | E 324 | E 324 | E 284 | E 287 | E 322 | E 312 |

| | | | | | | | | | | |
|---|---|---|---|---|---|---|---|---|---|---|
| **ATP interaction** | G 104 | G 104 | G 104 | G 113 | G 113 | G 113 | G 74 | G 76 | G 111 | G 106 |
| | T 105 | T 105 | T 105 | T 114 | T 114 | T 114 | T 75 | T 77 | T 112 | T 107 |
| | N 106 | N 106 | N 106 | N 114 | N 114 | N 114 | N 76 | N 78 | N 113 | **S 108** |
| | S 177 | S 170 | S 170 | S 186 | S 186 | S 186 | S 143 | S 149 | S 184 | S 173 |
| | K 195 | K 188 | K 188 | K 204 | K 204 | K 204 | K 161 | K 167 | K 202 | K 191 |
| | D 230 | D 223 | D 223 | D 239 | D 239 | D 239 | D 196 | D 202 | D 236 | **N 226** |
| | T 253 | **A 246** | **A 246** | T 262 | T 262 | T 262 | T 219 | T 225 | T 260 | **A 249** |
| | G 254 | G 247 | G 247 | G 263 | G 263 | G 263 | G 220 | G 226 | G 261 | G 250 |
| | D 439 | **E 438** | **E 438** | D 450 | D 450 | D 449 | D 406 | D 409 | D 445 | **E 444** |
| | G 440 | G 439 | G 439 | G 451 | G 451 | G 450 | G 407 | G 410 | G 446 | G 445 |
| | G 441 | G 440 | G 440 | G 452 | G 452 | G 451 | G 408 | G 411 | G 447 | G 446 |
| | S 478 | S 477 | S 477 | S 489 | S 489 | S 486 | S 445 | S 448 | S 484 | S 483 |
| **Conserved Glycine** | G 91 | G 91 | G 91 | G 100 | G 100 | G 100 | G 61 | G 63 | G 98 | G 93 |
| | G 95 | G 95 | G 95 | G 104 | G 104 | G 104 | G 65 | G 67 | G 102 | G 97 |
| | G 103 | G 103 | G 103 | G 112 | G 112 | G 112 | G 73 | G 75 | G 110 | G 105 |
| | G 173 | G 166 | G 166 | G 182 | G 182 | G 182 | G 139 | G 145 | G 180 | G 169 |
| | G 252 | G 245 | G 245 | G 261 | G 261 | G 261 | G 218 | G 224 | G 259 | G 248 |
| | G 254 | G 247 | G 247 | G 263 | G 263 | G 263 | G 220 | G 226 | G 261 | G 250 |
| | G 310 | **N 303** | **N 303** | G 319 | G 319 | G 319 | G 279 | G 282 | **D 317** | **Y 307** |
| | G 320 | G 313 | G 313 | G 329 | G 329 | G 329 | G 289 | G 292 | G 327 | G 317 |
| | G 440 | G 439 | G 439 | G 451 | G 451 | G 450 | G 407 | G 410 | G 446 | G 445 |
| | G 479 | G 478 | G 478 | G 490 | G 490 | G 489 | G 446 | G 449 | G 485 | **V 484** |

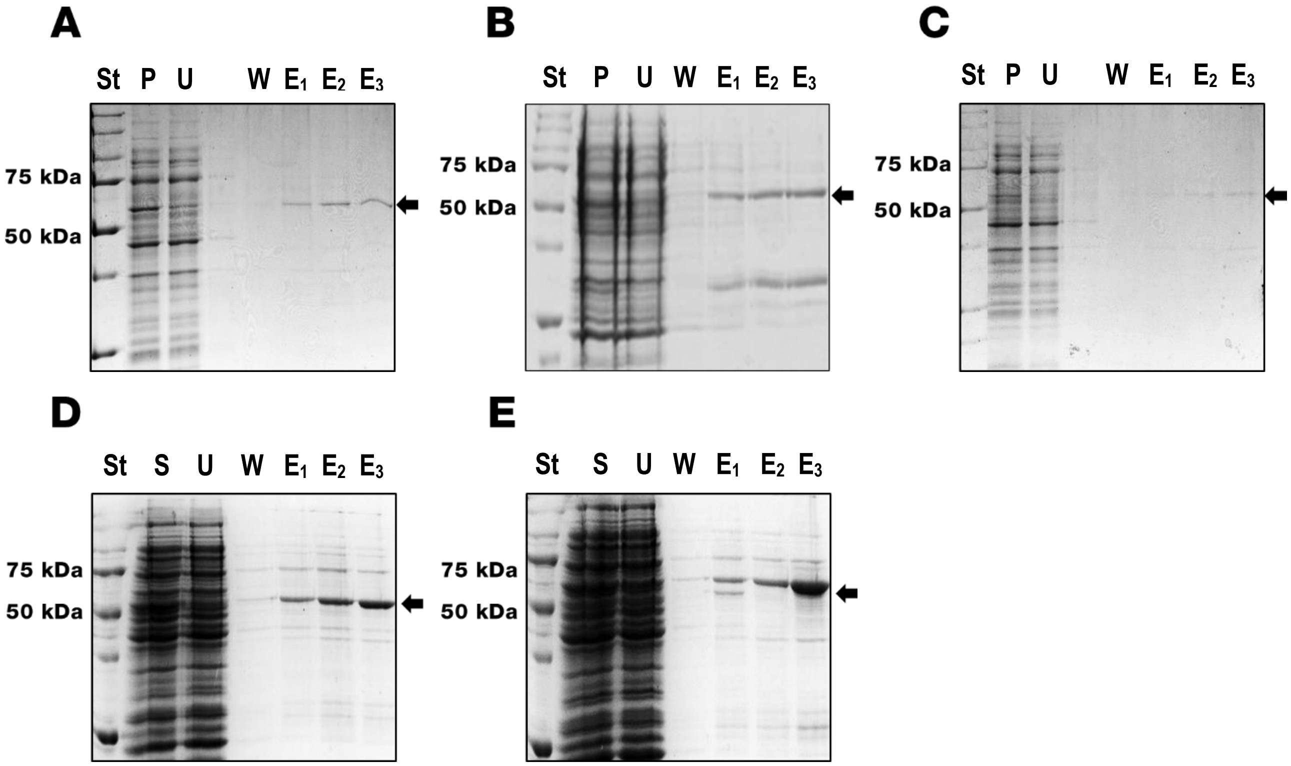


**Fig S2.** SDS-PAGE of full versions of recombinant ZmHXKs. Purification process of recombinant: **(A)** ZmHXK4, **(B)** ZmHXK5, **(C)** ZmHXK6, **(D)** ZmHXK7, and **(E)** ZmHXK8. St: Molecular weight standard, P: Pellet clarified with urea, S: Soluble supernatant, U: Unbound, W: Wash, $E_X$: Elution.

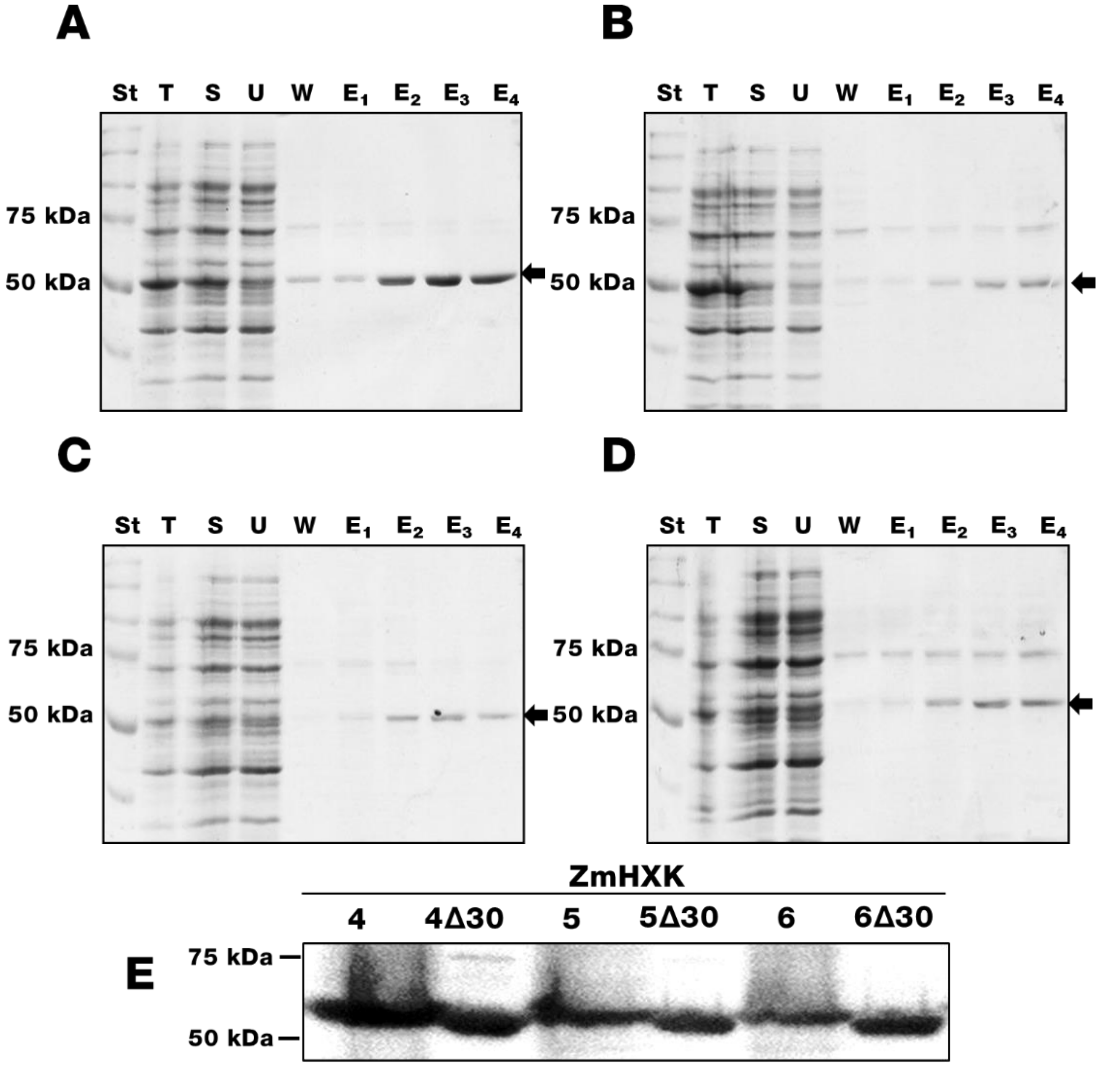


**Fig S3.** Purification of truncated versions of recombinant ZmHXKs**.** Purification process of recombinant: **(A)** ZmHXK4Δ30, **(B)** ZmHXK5Δ30, **(C)** ZmHXK6Δ30 and **(D)** ZmHXK9Δ30. All fractions were separated by SDS-PAGE and stained with Coomasie blue. **(E)** Western Blot of full and truncated versions of ZmHXK4-6**.** The proteins were detected using anti-V5-HRP. St: Molecular weight standard, T: Total cell extract S: Soluble supernatant, U: Unbound, W: Wash, $E_X$: Elution.

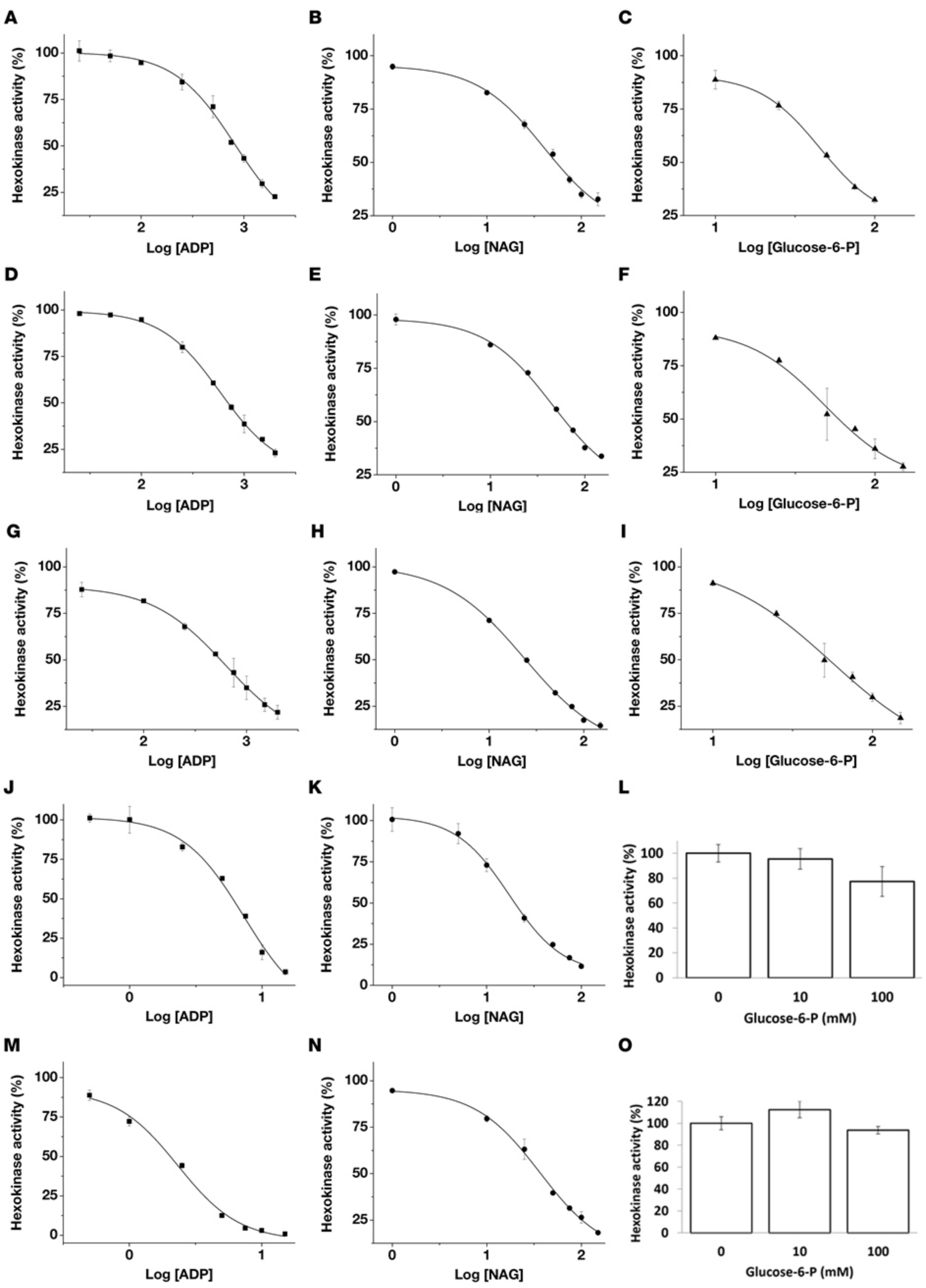


**Fig S4**. Inhibitory effects of ADP, NAG and G6P on ZmHXKs. (**A, B, C**) ZmHXKΔ4, (**D, E, F**) ZmHXKΔ5, (**G, H, I**) ZmHXKΔ6, (**J, K, L**) ZmHXK7, and (**M, N, O**) ZmHXK8.

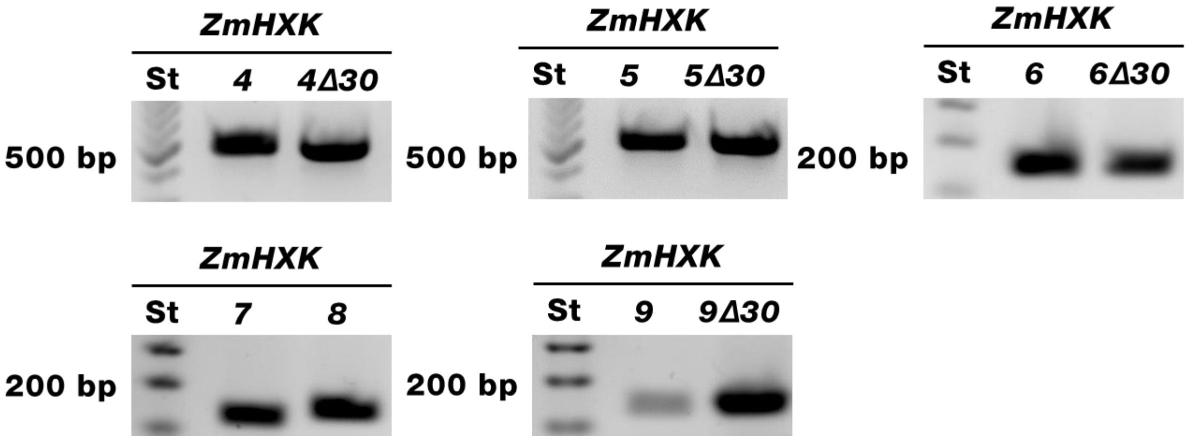


**Fig S5.** Expression profile of full and truncated versions of ZmHXKs in the JT 20088 yeast mutant. St: Molecular weight standard.

**Table S3.** Subcellular prediction of ZmHXKs.

| Protein | Transmembrane domain | Transit peptide | Subcellular localization |
|---|---|---|---|
| **ZmHXK3a** | No | 5-25 | Mitochondrion |
| **ZmHXK3b** | No | 5-25 | Mitochondrion |
| **ZmHXK4** | 4-24 | No | Secretory pathway |
| **ZmHXK5** | 4-24 | No | Secretory pathway |
| **ZmHXK6** | 9-29 | No | Secretory pathway |
| **ZmHXK7** | No | No | Other |
| **ZmHXK8** | No | No | Chloroplast |
| **ZmHXK9** | 4-24 | No | Secretory pathway |
| **ZmHXK10** | 6-26, 339-359 | No | Secretory pathway |

The prediction of transmembrane domains and transit peptide was made with the full amino acid sequence of each HXK with the aid of Toppred 1.10 software. The first 60 amino acids of each HXK protein sequence was used to predict the subcellular localization with the TargetP 1.1 software [32].

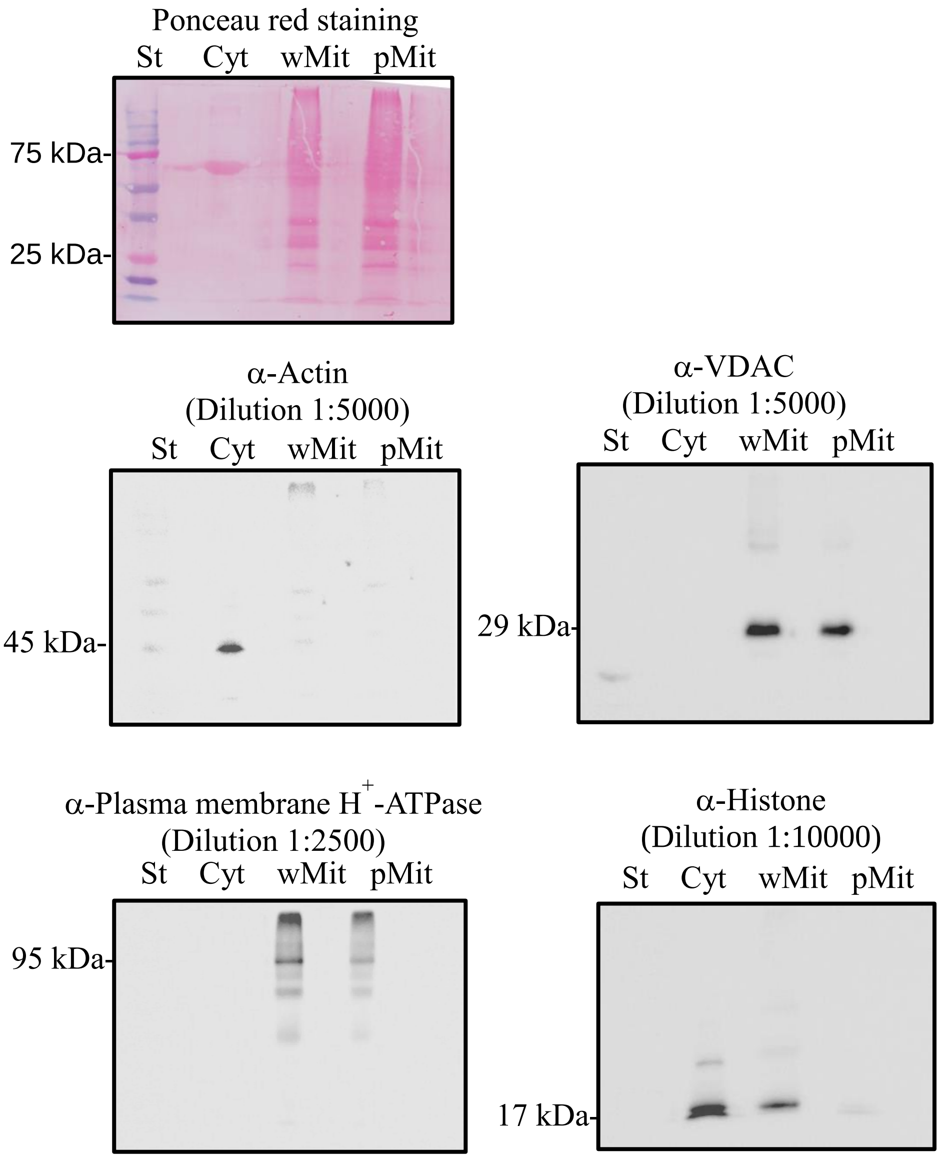


**Fig S6.** Evaluation of cytosolic and mitochondrial purity using specific antibodies. The purity of the cytosolic (Cyt), mitochondrial washed (wMit) and mitochondrial Percoll purified (pMit) fractions (10 µg) was evaluated by Western blot using Agrisera (Vännäs, Sweden) antibodies. These are representative membranes of at least three replicates.

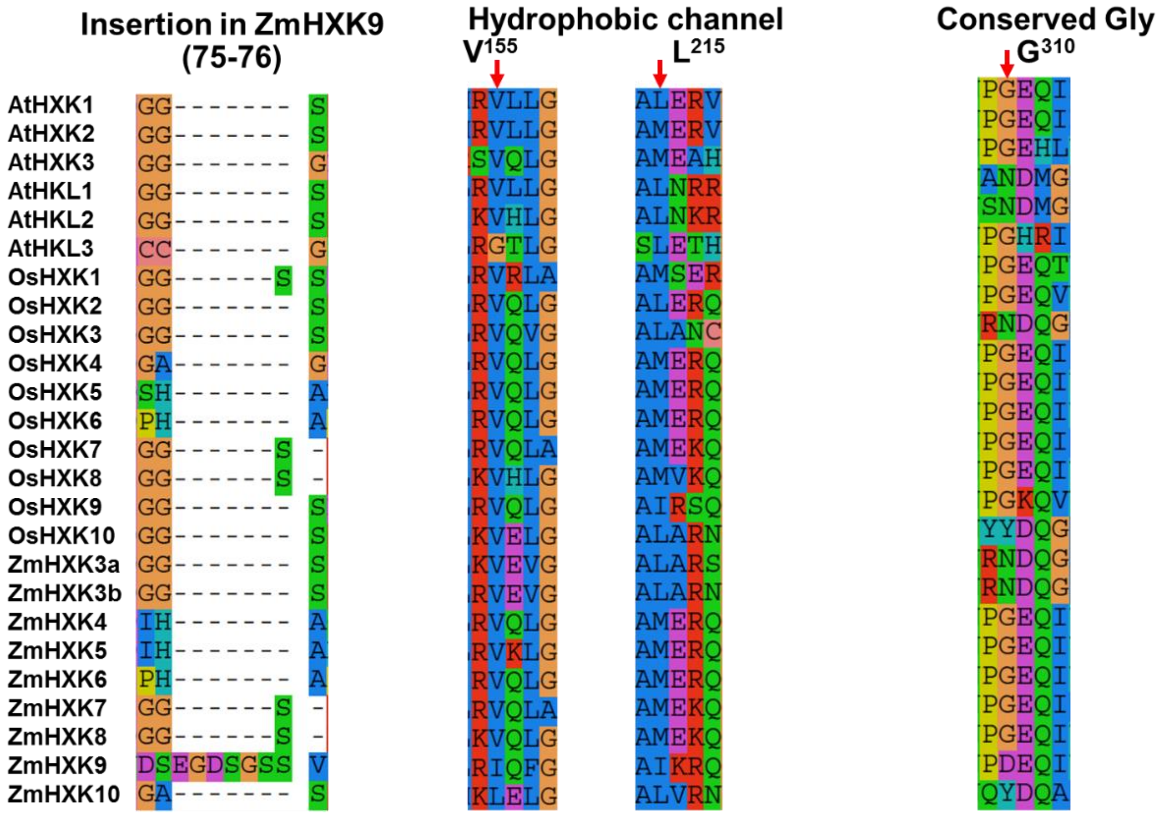


**Fig S7.** Changes in the amino acids of ZmHXK9 that could explain its low activity. The sequences were aligned using SeaView 4 [32].

**Table S4.** List of primers used for qPCR analysis, subcloning each maize HXK and PCR analysis in the yeast mutant.

| | |
|---|---|
| **qPCR primers** | |
| *ZmHXK4-RT-Fw* | GTGATCGAGGAGGTCGAGAG |
| *ZmHXK4-RT-Rv* | AACAGCCCATGTTCATCTCC |
| *ZmHXK5-RT-Fw* | GTGTGCTGCGAGTCCAACTA |
| *ZmHXK5-RT-Rv* | GGAAGGAAAACGTGAAACCA |
| *ZmHXK6-RT-Fw* | CATTGCTGCTGAGTTGGAAA |
| *ZmHXK6-RT-Rv* | CTTTCCATGGCCCTACTCAA |
| *ZmHXK7-RT-Fw* | CTGGTTTCGGGCATGTATCT |
| *ZmHXK7-RT-Rv* | GACCACCATCTTCCTCGTGT |
| *ZmHXK8-RT-Fw* | TGCTCCTCTCCTACGTCGAT |
| *ZmHXK8-RT-Rv* | GCCGACTTCTCTGGAGTCAC |
| *ZmHXK9-RT-Fw* | TTCAGTTGCATCTGGCACTC |
| *ZmHXK9-RT-Rv* | CATATCTCCCAGCAGCCAAT |
| **Subcloning primers and adapters** | |
| **(**Bases necessary for recombination and not present in original template are underlined) | |
| *AtHXK1-GW-Fw* | AAAAAGCAGGCTTCGAAGGAGATAGAACCATGGGTAAAGTAGCTGTTGGAG |
| *AtHXK1-GW-Rv* | AGAAAGCTGGGTGAGAGTCTTCAAGGTAGAGAGAGTG |
| *ZmHXK4-GW-Fw* | AAAAAGCAGGCTTCGAAGGAGATAGAACCATGGTGAAGGCCGTGGTGGT |
| *ZmHXK4-GW-Rv* | AGAAAGCTGGGTGGTCACTCGCGCCATACTGATACTG |
| *ZmHXK5-GW-Fw* | AAAAAGCAGGCTTCGAAGGAGATAGAACCATGGGGAAGTCCGTGGTGGT |
| *ZmHXK5-GW-Rv* | AGAAAGCTGGGTGGTCACTCTCGCCATACTGGGAGT |
| *ZmHXK6-GW-Fw* | AAAAAGCAGGCTTCGAAGGAGATAGAACCATGGCGAAGGGCGGTGCGGT |
| *ZmHXK6-GW-Rv* | AGAAAGCTGGGTGTGCAGCTTCAGCATACTGGGAGTGC |
| *ZmHXK7-GW-Fw* | AAAAAGCAGGCTTCGAAGGAGATAGAACCATGGTGGCGGCGGCGGAC |
| *ZmHXK7-GW-Rv* | AGAAAGCTGGGTGGTACTGCGAGTGGGCAGCTGCAA |

| | | |
|---|---|---|
| *ZmHXK8-GW-Fw* | AAAAAGCAGGCTTCGAAGGAGATAGAACCATGGCGGCAGCTGCGCTGG | |
| *ZmHXK8-GW-Rv* | AGAAAGCTGGGTGCTGAGATTGCGAGGCAGCAATCAGGG | |
| *ZmHXK9-GW-Fw* | AAAAAGCAGGCTTCGAAGGAGATAGAACCATGCGGAAGCCGGCGGCACT | |
| *ZmHXK9-GW-Rv* | AGAAAGCTGGGTGATCATCCACGGCCTGGAGACGTTG | |
| *ZmHXK4Δ30-GW-Fw* | AAAAAGCAGGCTTCGAAGGAGATAGAACCATGGACGCCGACCTCCTGGGG | |
| *ZmHXK5Δ30-GW-Fw* | AAAAAGCAGGCTTCGAAGGAGATAGAACCATGGACGCCGCGCTCCTGGG | |
| *ZmHXK6Δ30-GW-Fw* | AAAAAGCAGGCTTCGAAGGAGATAGAACCATGAGGAGGAGTCGGCGGCGG | |
| *ZmHXK9Δ30-GW-Fw* | AAAAAGCAGGCTTCGAAGGAGATAGAACCATGGCGAGGCGATGGGCACGC | |
| **Primers for the PCR analysis in the yeast mutant** | | |
| | | **Size (bp)** |
| *ZmHXK4-Fw* | CACGGTTGGTGAAGATGTTG | 480 |
| *ZmHXK4-Rv* | TGACATATCTGGCGTCCTCA | |
| *ZmHXK5-Fw* | CACGGTTGGTGAAGATGTTG | 480 |
| *ZmHXK5-Rv* | TGACATATCTGGCGTCCTCA | |
| *ZmHXK6-RT-Fw* | GGGACTCTAATTAAGTGGACCAAAG | 147 |
| *ZmHXK6-RT-Rv* | AGCCAATGTGCCTACAGTATC | |
| *ZmHXK7-RT-Fw* | GCTGTTGGTGAGGATGTCG | 130 |
| *ZmHXK7-RT-Rv* | CGTCCTCATCGTTGTACCG | |
| *ZmHXK8-RT-Fw* | TCTCCTACGTCGATAAGCTCC | 180 |
| *ZmHXK8-RT-Rv* | GAATGCCGACTTCTCTGGAG | |
| *ZmHXK9-RT-Fw* | GAAATTGTCCGAAGGGTCTTG | 137 |
| *ZmHXK9-RT-Rv* | TCAGGCGATGTGTCTTGATG | |
